# Customizable Stochastic High-Fidelity Model of the Sensors and Camera Onboard a Fixed Wing Autonomous Aircraft

**DOI:** 10.3390/s22155518

**Published:** 2022-07-24

**Authors:** Eduardo Gallo, Antonio Barrientos

**Affiliations:** Centro de Automática y Robótica, Universidad Politécnica de Madrid-Consejo Superior de Investigaciones, c/José Cutiérrez Abascal 2, 28006 Madrid, Spain; antonio.barrientos@upm.es

**Keywords:** aircraft sensors, IMU model, camera model, simulation, pseudo-random

## Abstract

The navigation systems of autonomous aircraft rely on the readings provided by a suite of onboard sensors to estimate the aircraft state. In the case of fixed wing vehicles, the sensor suite is usually composed by triads of accelerometers, gyroscopes, and magnetometers, a Global Navigation Satellite System (GNSS) receiver, and an air data system (Pitot tube, air vanes, thermometer, and barometer), and it is often complemented by one or more digital cameras. An accurate representation of the behavior and error sources of each of these sensors, together with the images generated by the cameras, is indispensable for the design, development, and testing of inertial, visual, or visual–inertial navigation algorithms. This article presents realistic and customizable models for each of these sensors; a ready-to-use C++ implementation is released as open-source code so non-experts in the field can easily generate realistic results. The pseudo-random models provide a time-stamped series of the errors generated by each sensor based on performance values and operating frequencies obtainable from the sensor’s data sheets. If in addition, the simulated true pose (position plus attitude) of the aircraft is provided, the camera model generates realistic images of the Earth’s surface that resemble those taken with a real camera from the same pose.

## 1. Introduction

**Figure 1:**
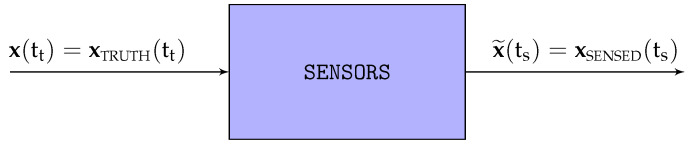
Sensors flow diagram.

The sensors onboard an autonomous aircraft measure various aspects of the aircraft *real* or *actual state* $x=x_{\mathrm{TRUTH}}$ and provide these measurements to the aircraft guidance, navigation, and control (GNC) system. The outputs of these sensors, collectively known as the *sensed state* $\tilde{x}=x_{\mathrm{SENSED}}$, represent the only link between the real but unknown actual states and the GNC system in charge of achieving an actual trajectory that deviates as little as possible from the guidance targets (Figure 1).

Researchers and engineers designing, developing, or testing aircraft navigation systems require realistic renditions of the time variations of both the actual and sensed states to analyze the behavior of their algorithms in simulation. It is only after validating the algorithms under a wide range of conditions that these can be installed onboard the aircraft and field tested. The obtainment of realistic $x=x_{\mathrm{TRUTH}}$ states covering the different maneuvers to be analyzed is generally achieved by discrete integration of the aircraft equations of motion coupled with the applicable guidance targets. Realistic $\tilde{x}=x_{\mathrm{SENSED}}$ sensor outputs are however quite difficult to generate given their stochastic nature, the various underlying technologies, and the lack of detailed error models from the sensor manufacturers. In addition, realistic-looking images of the Earth’s surface that resemble those taken from a real aircraft are also necessary to test visual navigation algorithms.

Note that although the actual aircraft state varies continuously in the real world, in simulation, it is usually the outcome of a high-frequency discrete integration process [1] that results in $x\left(t_{t}\right)=x_{\mathrm{TRUTH}}\left(t_{t}\right)$, where $t_{t}=t\cdot \Delta t_{\mathrm{TRUTH}}$. The sensed trajectory $\tilde{x}\left(t_{s}\right)=x_{\mathrm{SENSED}}\left(t_{s}\right)$, where $t_{s}=s\cdot \Delta t_{\mathrm{SENSED}}$, is however intrinsically discrete, although the working frequency of the different sensors may vary. This article considers that all sensors operate at the same rate of $\Delta t_{\mathrm{SENSED}}$, with the exception of the GNSS receiver and the onboard camera, which work at $\Delta t_{\mathrm{GNSS}}$ and $\Delta t_{\mathrm{IMG}}$, respectively. It is also assumed that all sensors are fixed to the aircraft structure in a strapdown configuration and that their measurement processes are instantaneous and time synchronized with each other at their respective frequencies.

The sensed states or sensed trajectory can be defined as a time-stamped series of state vectors $\tilde{x}=x_{\mathrm{SENSED}}$ that groups the measurements provided by the different onboard sensors (1) (note that when present, the super index represents the frame or reference system in which a certain variable is viewed; if two sub-indexes are present, it implies that the vector goes from the first frame to the second. For example, $\omega_{\mathrm{IB}}^{B}$ represents the angular velocity from the inertial frame $F_{I}$ to the body frame $F_{B}$ viewed in body. This article makes use of the body frame $F_{B}$, which is rigidly attached to the aircraft structure with origin in its center of mass [2], the NED frame $F_{N}$ also centered on the aircraft center of mass with axes in the North–East–Down directions [2], and the inertial frame $F_{I}$, which is usually considered as centered in the Sun with axes fixed with respect to other stars [3]), comprising the only view of the actual states at the disposal of the navigation system. Its components are listed in Table 1. Note that the *specific force* $f_{\mathrm{IB}}$ is defined as the non-gravitational acceleration experienced by the aircraft body with respect to an inertial frame [4].

(1) $$\tilde{x}=x_{\mathrm{SENSED}}={\left[{\tilde{f}}_{\mathrm{IB}}^{B},\;{\tilde{\omega }}_{\mathrm{IB}}^{B},\;{\tilde{B}}^{B},\;{\tilde{x}}_{\mathrm{GDT}},\;{\tilde{v}}^{N},\;\tilde{p},\;\tilde{T},\;{\tilde{v}}_{\mathrm{TAS}},\;\tilde{\alpha },\;\tilde{\beta },\;\;I\right]}^{T}$$

Following a review of the objectives, state of the art, and novelty in Section 1.1, Section 1.2, Section 1.3, the following sections provide detailed descriptions of the stochastic models representing the errors present in the measurements of the different sensors: Section 2 describes the inertial sensors (accelerometers and gyroscopes), Section 3 focuses on the magnetometers, GNSS receiver, and air data system, and Section 4 presents the tool employed to generate realistic images that resemble what a real camera would view if located at the same position and attitude. Although the camera differs from all other sensors in that it does not provide a measurement or reading but a digital image, in this article, it is indeed considered a sensor as it provides the navigation system with information about its surroundings that can be employed for navigation. Section 5 describes sensor calibration activities that are indispensable for the determination of various parameters present in the sensor models. Section 6 discusses the main characteristics of the models, with special emphasis on the input seeds that control its stochastic properties; it also includes an example on how to customize the models for the case of a low Size, Weight, and Power (SWaP) aircraft. The conclusions are presented in Section 7.

### 1.1. Objectives

Sensor manufacturers do not provide models with which to estimate the errors introduced by their products, this is, the differences between the actual and sensed states. Instead, they usually publish data sheets that contain selected performance parameters in different formats and units, with few or no instructions for their interpretation. In the case of high-grade inertial sensors, an Allan variance curve is sometimes provided.

Faced with this situation, researchers willing to understand how their GNC systems will perform when the aircraft is equipped with given sensors face a difficult choice, especially in the case of low-cost sensors. One possibility is to adopt a sensor model from the literature and rely on the Allan variance curve (if available) to identify the required parameters, although it is not always clear how to do so [5]. If the Allan curve is not available, a second possibility is to obtain the curve by analyzing the sensor outputs when placed on a test bench, but the process is time consuming, requires specialized equipment and know-how, and the results are only valid for the specific tested hardware [5]. The end result is that researchers often rely on simple sensor models, which although easy to implement, fail to provide realistic outputs of the errors introduced by each sensor type. This has negative consequences for the performance of their GNC algorithms, which may not work as desired when faced with the real sensor outputs instead of the simulated ones employed for their development.

The first objective of this article is to address the need for realistic error models that can be quickly customized by the user based exclusively on the performance parameters contained in the data sheets provided by the sensor manufacturers, without the need for specific test equipment nor expert knowledge in the behavior of the different sensors. By implementing the described models or employing the provided open-source C++ code [6], the end user can quickly obtain realistic pseudo-random results without any expertise in the behavior of the different onboard sensors. Once the user introduces the desired performance parameters and operating frequencies, the simulated outputs of all onboard sensors rely exclusively on two input seeds (one identifying the airframe and the other identifying the specific flight). Different pairs of seeds can be employed as part of a Monte Carlo simulation, or the same pair can be used repeatedly in case the same outputs are required for further analysis. The use of the proposed models hence enables researchers to quickly obtain realistic time-stamped series of the values of $\tilde{x}=x_{\mathrm{SENSED}}$ with which to feed their simulations.

The second objective of this article is to develop a camera model capable of generating realistic images, resembling what a real camera would record if mounted on the aircraft, so the resulting images of the Earth’s surface can be employed for the development and testing of visual and visual–inertial navigation systems. The resulting Earth Viewer application, described in Section 4.2, is also released as open-source code within [6].

### 1.2. State of the Art

The standard means for conveying the performance of a given single-axis inertial sensor is by means of its Allan variance curve [5], even though manufacturers do not always make it available, in particular for low-cost sensors. Although standards exist for how to generate the curve [7,8,9], it is not always clear how to convert the Allan variance information into a suitable sensor model [5]. Various Allan curve translation methods [10,11,12,13,14,15,16,17,18,19] have been available for a long time, but [5] provides the first clear exposition of the underlying ideas, issues, and trade-offs between the different methods. More recent attempts to identify the required parameters involve the use of maximum likelihood estimators [20] as well as machine learning [21].

As described in [5], the Allan variance is a well-known time domain analysis technique originally developed to analyze the frequency stability of oscillators [22,23,24], which has been successfully adopted to communicate the performances of inertial sensors and to characterize their stochastic errors [11,13,15,18,19,25,26,27,28,29].

The various error sources that influence the output of an inertial sensor are described in multiple navigation textbooks, such as [2,4,30,31,32,33]. In addition to the single-axis sensor model described in [5], the system noise and random walk contributions of the final error of a single-axis sensor are discussed in detail in [34,35], which constitute the basis for the model presented in Section 2.2. The added difficulty of combining three inertial sensors into a triad, treated in Section 2.5, is discussed in [2,31,32]. Basic magnetometer and GNSS receiver models can also be found in these navigation textbooks.

### 1.3. Novelty

The main contribution of this article is that it provides customizable, stochastic, and realistic models for the errors introduced by the various sensors onboard a fixed wing aircraft, with special emphasis on the inertial ones (accelerometers and gyroscopes), without relying on the Allan variance curves, as is the case in the rest of the literature reviewed in Section 1.2. The required characteristics of the models are the following:

- *Customizable* so the user can employ the values that better resemble the performances of the specific equipment being modeled.
- *Stochastic* to properly represent the nature of the different random processes involved, while ensuring that the time variation of the errors generated by each sensor can be repeated if so desired.
- *Realistic* to provide a faithful description of the variation with time of the measurement errors, including as few simplifications as possible.

This enables researchers to quickly generate pseudo-random time-stamped series of the errors introduced by each sensor without the need for the expert know-how in the behavior of each sensor required to process the Allan variance curve nor the expensive and time-consuming process required to generate the curve independently. The results can be employed to feed Monte Carlo simulations that require the sensor readings as inputs, such as those required to analyze the behavior of GNC algorithms.

To develop comprehensive models whose parameters can be obtained exclusively from the data sheets published by the manufacturers, the authors have built on established models for the system noise and random walk contributions to single-axis sensors as well as the scale factor and cross-coupling contributions that appear when using sensor triads. The comprehensive models take into consideration the influence of the true relative pose (position plus attitude) of the sensor triad with respect to the platform as well as the uncertainty in the processor’s knowledge about such poses. The contribution of the various calibration procedures on the required parameters is also discussed.

The second contribution of this article is the release of the Earth Viewer application, which is capable of providing realistic and distortion-free images of the Earth surface that resemble what a real camera would record when mounted on the aircraft. To the knowledge of the authors, this is the first time that a tool capable of considering the six degrees of freedom of the camera pose has been published. These images can be employed to test the behavior of visual and visual inertial navigation systems.

## 2. Inertial Sensors

Inertial sensors comprise *accelerometers* and *gyroscopes*, which measure the specific force and inertial angular velocity about a single axis, respectively [36]. An *inertial measurement unit* (IMU) encompasses multiple accelerometers and gyroscopes, usually three of each, obtaining three-dimensional measurements of the specific force and angular rate [2] viewed in the platform frame $F_{P}$ (Section 2.5). However, the individual accelerometers and gyroscopes are not aligned with the $F_{P}$ axes but with those of the non-orthogonal accelerometers $F_{A}$ and gyroscopes $F_{Y}$ frames, which are also defined in Section 2.5. The output of the inertial sensors must hence first be transformed from the $F_{A}$ and $F_{Y}$ frames to $F_{P}$, as described in Section 2.6 and Section 2.7, and then from the $F_{P}$ frame to the body frame $F_{B}$ as explained in Section 2.8, where they can be employed by the navigation system. The accelerometers and gyroscopes are assumed to be infinitesimally small and located at the IMU reference point (Section 2.8), which coincides with the origin of these three frames $\left(O_{P}=O_{A}=O_{Y}\right)$.

The IMU is physically attached to the aircraft structure in a strapdown configuration, so both the displacement $T_{\mathrm{BP}}^{B}$ and the Euler angles $\phi^{\mathrm{BP}}={\left[\psi^{P},\;\theta^{P},\;\xi^{P}\right]}^{T}$ that describe the relative position and rotation between the body $F_{B}$ and platform $F_{P}$ frames are constant. Accelerometers can be divided by their underlying technology into pendulous and vibrating beams, while gyroscopes are classified into spinning mass, optical (ring laser or fiber optic), and vibratory [4]. Current inertial sensor development is mostly focused on *micro machined electromechanical system* (MEMS) sensors (there exist both pendulous and vibrating beam MEMS accelerometers, but all MEMS gyroscopes are vibratory), which makes direct use of the chemical etching and batch processing techniques used by the electronics integrated circuit industry to obtain sensors with small size, low weight, rugged construction, low power consumption, low price, high reliability, and low maintenance [30]. On the negative side, the accuracy of MEMS sensors is still low, although tremendous progress has been achieved in the last two decades, and more is expected in the future.

There is no universal classification of inertial sensors according to their performance, although they can be broadly assigned into five different categories or grades: marine (submarines and spacecraft), aviation (commercial and military), intermediate (small aircraft and helicopters), tactical (unmanned air vehicles and guided weapons), and automotive (consumer) [4]. The full range of grades covers approximately six orders of magnitude of gyroscope performance and only three for the accelerometers, but higher performance is always associated with bigger size, weight, and cost. Tactical grade IMUs cover a wide range of performance values but can only provide a stand-alone navigation solution for a few minutes, while automotive grade IMUs are unsuitable for navigation.

The different errors that appear in the measurements provided by accelerometers and gyroscopes are described in Section 2.1. Section 2.2 presents a model for the measurements of a single inertial sensor, while Section 2.3 and Section 2.4 focus on how to obtain the specific values for white noise and bias on which the model relies from the documentation. Section 2.5 describes the reference systems required to represent the IMU measurements. Additional errors appear when three accelerometers or gyroscopes are employed together, and these are modeled in Section 2.6 for accelerometers and Section 2.7 for gyroscopes. The analysis of the inertial sensors concludes with Section 2.9, which provide a comprehensive error model for the IMU measurements. The final model also depends on the relative position of the IMU with respect to the body frame, which is described in Section 2.8.

### 2.1. Inertial Sensor Error Sources

In addition to the accelerometers and gyroscopes, an IMU also contains a processor, storage for the calibration parameters, one or more temperature sensors, and a power supply. As described below, each sensor has several error sources, but each of them has four components: *fixed* contribution, *temperature*-dependent variation, *run-to-run* variation, and *in-run* variation [4,37]. The first two can be measured at the laboratory (at different temperatures) and the calibration results can be stored in the IMU so the processor can later compensate the sensor outputs based on the reading provided by the temperature sensor. Calibration, however, increases manufacturing costs, so it may be absent in the case of inexpensive sensors. The run-to-run variation results in a contribution to a given error source that varies every time the sensor is employed but remains constant within a given run. It cannot be compensated by the IMU processor but can be calibrated by the navigation system every time it is turned on with a process known as fine alignment [2,4,31]. The in-run contribution to the error sources slowly varies during execution and cannot be calibrated in the laboratory nor by the navigation system.

Let us now discuss the different sources of error that influence an inertial sensor [4,38,39,40]:

- The *bias* is an error exhibited by all accelerometers and gyroscopes that is independent of the underlying specific force or angular rate being measured, and it comprises the dominant contribution to the overall sensor error. It can be defined as any nonzero output when the sensor input is zero [37], and it can be divided into its static and dynamic components. The static part, also known as fixed bias, *bias offset*, turn-on bias, or bias repeatability, comprises the run-to-run variation, while the dynamic component, known as in-run bias variation, *bias drift*, or bias instability (or stability), is typically about 10% of the static part and slowly varies over periods of the order of one minute. As the bias is the main contributor to the overall sensor error, its value can be understood as a sensor quality measure. Table 2 provides approximate values for the inertial sensor biases according to the IMU grade [4].
  While the bias offset can be greatly reduced through fine alignment [2,4,31], the bias drift cannot be determined and needs to be modeled as a stochastic process. It is mostly a warm-up effect that should be almost non-existent after a few minutes of operation, and it corresponds to the minimum point of the sensor’s Allan curve [7,8,9,39]. It is generally modeled as a random walk process obtained by the integration of a white noise signal coupled with limits that represent the conclusion of the warm up process.
- The *scale factor error* is the departure of the input output gradient of the instrument from unity following unit conversion at the IMU processor. It represents a varying relationship between sensor input and output caused by aging and manufacturing tolerances. As it is a combination of fixed contribution plus temperature-dependent variation, most of it can be eliminated through calibration (Section 5.1).
- The *cross-coupling error* or non-orthogonality error is a fixed contribution that arises from the misalignment of the sensitive axes of the inertial sensors with respect to the orthogonal axes of the platform frame due to manufacturing limitations, and it can also be highly reduced through calibration. The scale factor and cross-coupling errors are in the order of $10^{-4}$ and $10^{-3}$ for most inertial sensors, although they can be higher for some low-grade gyroscopes. The cross-coupling error is equal to the sine of the misalignment, which is listed by some manufacturers.
- *System noise* or random noise is inherent to all inertial sensors and can combine electrical, mechanical, resonance, and quantization sources. It can originate at the sensor itself or at any other electronic equipment that interferes with it. System noise is a stochastic process usually modeled as white noise because its noise spectrum is approximately white, and it cannot be calibrated as there is no correlation between past and future values. A white noise process is characterized by its power spectral density (PSD), which is constant as it does not depend on the signal frequency. It corresponds to the 1 s crossing of the sensor’s Allan curve [7,8,9,39].
  System noise is sometimes referred to as random walk, which can generate confusion with the bias. The reason is that the inertial sensor outputs are always integrated to obtain ground velocity in the case of accelerometers and aircraft attitude in the case of gyroscopes. As the integration of a white noise process is indeed a random walk, the later term is commonly employed to refer to system noise. Table 3 contains typical values for accelerometer and gyroscope root PSD according to sensor grade [4].
- Other minor error sources not considered in this article are the g-dependent bias (sensitivity of spinning mass and vibratory gyroscopes to specific force), scale factor nonlinearity, and higher-order errors (spinning mass gyroscopes and pendulous accelerometers).

### 2.2. Single-Axis Inertial Sensor Error Model

As the inertial sensors provide measurements at equispaced discrete times $t_{s}=s\cdot \Delta t_{\mathrm{SENSED}}=s\cdot \Delta t$, this section focuses on obtaining a discrete model for the bias and white noise errors of a single-axis inertial sensor. The results obtained here will be employed in the following sections to generate a comprehensive IMU model.

Let us consider a sensor in which the difference between its measurement at any given time $\tilde{x}\left(t\right)$ and the real value of the physical magnitude being measured at that same time $x\left(t\right)$ can be represented by a zero mean white noise Gaussian process $\eta_{v}\left(t\right)$ with spectral density $\sigma_{v}^{2}$:

(2) $$\tilde{x}\left(t\right)=x\left(t\right)+\eta_{v}\left(t\right)$$

Dividing (2) by $\Delta t_{\mathrm{SENSED}}=\Delta t$ and integrating results in:

(3) $$\frac{1}{\Delta t}\int_{t_{0}}^{t_{0}+\Delta t}\tilde{x}\left(t\right)\;\mathrm{dt}=\frac{1}{\Delta t}\int_{t_{0}}^{t_{0}+\Delta t}\left[x\left(t\right)+\eta_{v}\left(t\right)\right]\;\mathrm{dt}$$

Assuming that the measurement and real value are both constant over the integration interval (note that the stochastic process $\eta_{v}$ cannot be considered constant over any interval) [34] yields

(4) $$\tilde{x}\left(t_{0}+\Delta t\right)=x\left(t_{0}+\Delta t\right)+\frac{1}{\Delta t}\int_{t_{0}}^{t_{0}+\Delta t}\eta_{v}\left(t\right)\;\mathrm{dt}$$

This expression results in the white noise sensor error $w\left(t\right)$, which is the difference between the sensor measurement $\tilde{x}\left(t\right)$ and the true value $x\left(t\right)$. Its mean and variance can be readily computed:

(5) $$\begin{array}{ccc} w\left(t_{0}+\Delta t\right) & = & \frac{1}{\Delta t}\int_{t_{0}}^{t_{0}+\Delta t}\eta_{v}\left(t\right)\;\mathrm{dt} \end{array}$$

(6) $$\begin{array}{ccc} E\left[w\left(t_{0}+\Delta t\right)\right] & = & 0 \end{array}$$

(7) $$\begin{array}{ccc} \mathrm{Var}\left[w\left(t_{0}+\Delta t\right)\right] & = & \frac{\sigma_{v}^{2}}{\Delta t} \end{array}$$

Based on these results, the white noise error can be modeled by a discrete random variable identically distributed to the above continuous white noise error, that is, one that results in the same mean and variance, where $N_{v}∼N\left(0,\;1\right)$ is a standard normal random variable:

(8) $$w\left(s\;\Delta t_{\mathrm{SENSED}}\right)=w\left(s\;\Delta t\right)=\frac{\sigma_{v}}{\Delta t^{1/2}}\;N_{\mathrm{vs}}$$

Let us now consider a second model in which the measurement error or bias is given by a first-order random walk process or integration of a zero mean white noise Gaussian process $\eta_{u}\left(t\right)$ with spectral density $\sigma_{u}^{2}$:

(9) $$\dot{b}\left(t\right)=\eta_{u}\left(t\right)\;⟶\;b\left(t_{0}+\Delta t\right)=b\left(t_{0}\right)+\int_{t_{0}}^{t_{0}+\Delta t}\eta_{u}\left(t\right)\;\mathrm{dt}$$

Its mean and variance can be quickly computed:

(10) $$\begin{array}{ccc} E\left[b\left(t_{0}+\Delta t\right)\right] & = & E\left[b\left(t_{0}\right)\right] \end{array}$$

(11) $$\begin{array}{ccc} \mathrm{Var}\left[b\left(t_{0}+\Delta t\right)\right] & = & \sigma_{u}^{2}\;\Delta t \end{array}$$

These results indicate that the bias can be modeled by a discrete random variable identically distributed to the continuous random walk above:

(12) $$b\left(t_{0}+\Delta t\right)=b\left(t_{0}\right)+\sigma_{u}\;\Delta t^{1/2}\;N_{u}$$

where $N_{u}∼N\left(0,\;1\right)$ is a standard normal random variable. Operating with the above expression results in the final expression for the discrete bias as well as its mean and variance:

(13) $$\begin{array}{ccc} b\left(s\;\Delta t\right) & = & B_{0}\;N_{u0}+\sigma_{u}\;\Delta t^{1/2}\;\sum_{i=1}^{s}N_{\mathrm{ui}} \end{array}$$

(14) $$\begin{array}{ccc} E\left[b\left(s\;\Delta t\right)\right] & = & 0 \end{array}$$

(15) $$\begin{array}{ccc} \mathrm{Var}\left[b\left(s\;\Delta t\right)\right] & = & B_{0}^{2}+\sigma_{u}^{2}\;s\;\Delta t \end{array}$$

A comprehensive single-axis sensor error model without a scale factor can hence be constructed by adding together the influence of the system noise provided by (8) and the bias given by (13) [35], while assuming that the standard normal random variables $N_{u}$ and $N_{v}$ are uncorrelated (note that the expected value and variance of each of the two discrete components of this sensor model coincide with those of their continuous counterparts, but their combined mean and variance provided by expressions (17) and (18) differ from that of the combination of the two continuous error models given by (5) and (9). This is the case even if considering that the two zero mean white noise Gaussian processes $\eta_{u}$ and $\eta_{v}$ are independent and hence uncorrelated. It is however possible to obtain a discrete model whose discrete bias and white noise components are not only identically distributed to those of their continuous counterparts [34], even adding the equivalence of covariance between the bias and the sensor error, but this results in a significantly more complex model that behaves similarly to the one above at all but has the shortest time samples after sensor initialization. The authors have decided not to do so in the model described in this article, reducing complexity with little or no loss of realism):

(16) $$\begin{array}{ccc} e_{\mathrm{BW}}\left(s\;\Delta t\right) & = & \tilde{x}\left(s\;\Delta t\right)-x\left(s\;\Delta t\right)=B_{0}\;N_{u0}+\sigma_{u}\;\Delta t^{1/2}\;\sum_{i=1}^{s}N_{\mathrm{ui}}+\frac{\sigma_{v}}{\Delta t^{1/2}}\;N_{\mathrm{vs}} \end{array}$$

(17) $$\begin{array}{ccc} E\left[e_{\mathrm{BW}}\left(s\;\Delta t\right)\right] & = & 0 \end{array}$$

(18) $$\begin{array}{ccc} \mathrm{Var}\left[e_{\mathrm{BW}}\left(s\;\Delta t\right)\right] & = & B_{0}^{2}+\sigma_{u}^{2}\;s\;\Delta t+\frac{\sigma_{v}^{2}}{\Delta t} \end{array}$$

The *discrete sensor error* or difference between the measurement provided by the sensor $\tilde{x}\left(s\;\Delta t_{\mathrm{SENSED}}\right)=\tilde{x}\left(s\;\Delta t\right)$ at any given discrete time sΔt and the real value of the physical variable being measured at that same discrete time $x\left(s\;\Delta t\right)$ is the combination of a bias or first-order random walk and a white noise process, and it depends on three parameters: the bias offset $B_{0}$, the bias instability $\sigma_{u}$, and the white noise $\sigma_{v}$. The contributions of these three different sources to the sensor error as well as to its first and second integrals (gyroscopes measure angular velocity, and their output needs to be integrated once to obtain attitude, while accelerometers measure specific force and are integrated once to obtain velocity and twice to obtain position) are very different and inherent to many of the challenges encountered when employing accelerometers and gyroscopes for inertial navigation, as explained below.

**Figure 2:**
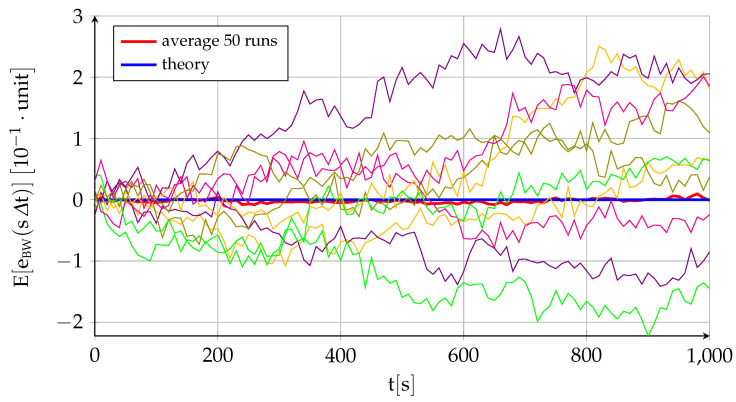
Propagation with time of sensor error mean.

**Figure 3:**
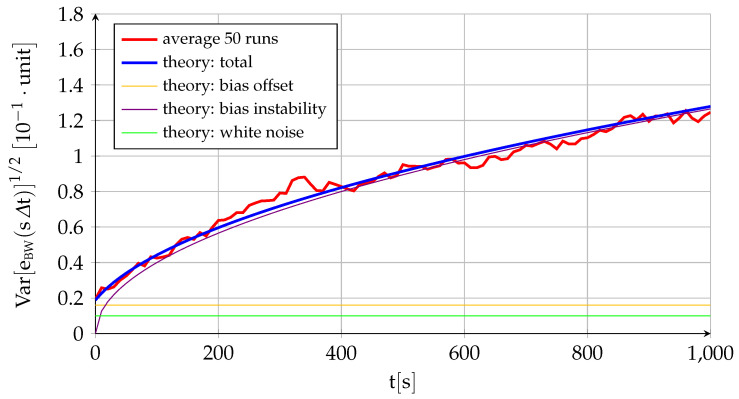
Propagation with time of sensor error standard deviation.

Figure 2 and Figure 3 represent the performance of a fictitious sensor of $B_{0}=1.6\times 10^{-2}$, $\sigma_{u}=4\times 10^{-3}$, and $\sigma_{v}=1\times 10^{-3}$ working at a frequency of $100\;\mathrm{Hz}\;\left(\Delta t=0.01\;s\right)$, and they are intended to showcase the different behavior and relative influence on the total error of each of its three components. The figures show the theoretical variation with time of the sensor error mean (Figure 2) and standard deviation (Figure 3) given by (17) and (18) together with the average of fifty different runs. In addition, Figure 2 also includes ten of those runs to showcase the variability in results implicit to the random variables (although the data are generated at 100Hz, for visibility purposes, the figure only employs 1 out of every 1000 points, so it appears far less noisy than the real data), while Figure 3 shows the theoretical contribution to the standard deviation of each of the three components. In addition to the near equivalence between the theory and the average of several runs, the figures show that the bias instability is the commanding long-term factor in the deviation between the sensor measurement and its zero mean (the standard deviation of the bias instability grows with the square root of time while the other two components are constant). As discussed in Section 2.1, the bias drift or bias instability is indeed the most important quality parameter of an inertial sensor. This is also the case when the sensor output is integrated, as discussed below.

Let us integrate the sensor error over a timespan sΔt to evaluate the growth with time of both its expected value and its variance (as the interest lies primarily in s≫1, a simple integration method such as the rectangular rule is employed):

(19) $$\begin{array}{ccc} f_{\mathrm{BW}}\left(s\;\Delta t\right) & = & f_{\mathrm{BW}}\left(0\right)+\int_{0}^{s\;\Delta t}e_{\mathrm{BW}}\left(\tau \right)\;d\tau =f_{\mathrm{BW}}\left(0\right)+\Delta t\sum_{i=1}^{s}e_{\mathrm{BW}}\left(i\;\Delta t\right)\\ & = & f_{\mathrm{BW}}\left(0\right)+B_{0}\;N_{u0}\;s\;\Delta t+\sigma_{u}\;\Delta t^{3/2}\sum_{i=1}^{s}\left(s-i+1\right)\;N_{\mathrm{ui}}+\\ & & +\sigma_{v}\;\Delta t^{1/2}\sum_{i=1}^{s}N_{\mathrm{vi}} \end{array}$$

(20) $$\begin{array}{ccc} E\left[f_{\mathrm{BW}}\left(s\;\Delta t\right)\right] & = & f_{\mathrm{BW}}\left(0\right)\\ \mathrm{Var}\left[f_{\mathrm{BW}}\left(s\;\Delta t\right)\right] & = & B_{0}^{2}\;{\left(s\;\Delta t\right)}^{2}+\frac{\sigma_{u}^{2}}{6}\;\Delta t^{3}\;s\;\left(s+1\right)\;\left(2\;s+1\right)+\sigma_{v}^{2}\;\left(s\;\Delta t\right) \end{array}$$

(21) $$\begin{array}{ccc} & \approx & B_{0}^{2}\;{\left(s\;\Delta t\right)}^{2}+\frac{\sigma_{u}^{2}}{3}\;{\left(s\;\Delta t\right)}^{3}+\sigma_{v}^{2}\;\left(s\;\Delta t\right) \end{array}$$

**Figure 4:**
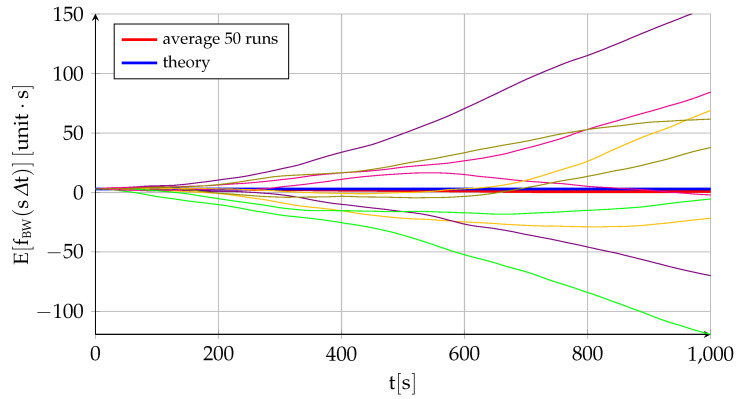
Propagation with time of first integral of sensor error mean.

**Figure 5:**
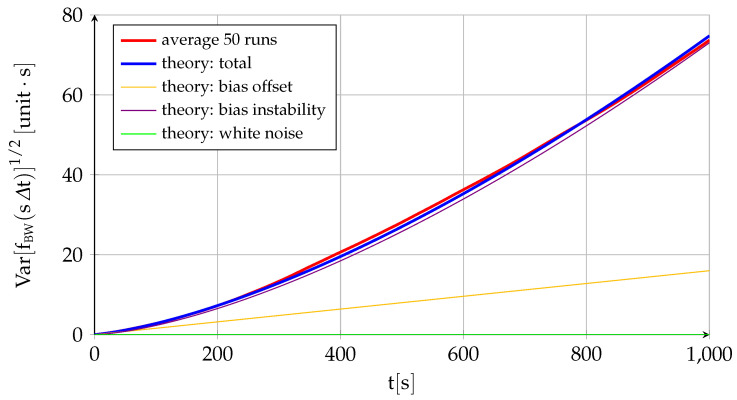
Propagation with time of first integral of sensor error standard deviation.

Figure 4 and Figure 5 follow the same pattern as Figure 2 and Figure 3 but applied to the error integral instead of to the error itself. They would represent the attitude error resulting from integrating the gyroscope output or the velocity error expected when integrating the specific force measured by an accelerometer. The conclusions are the same as before but significantly more accentuated. Not only is the expected value of the error constant instead of zero ($f_{\mathrm{BW}}\left(0\right)=3$ has been employed in the experiment), but the growth in the standard deviation (over a nonzero mean) is much quicker than before. The bias instability continues to be the dominating factor but now increases with a power of $t^{3/2}$, while the bias offset and white noise contributions also increase with time, although with powers of t and $t^{1/2}$, respectively. Let us continue the process and integrate the error a second time:

(22) $$\begin{array}{lll} g_{\mathrm{BW}}\left(s\;\Delta t\right) & = & g_{\mathrm{BW}}\left(0\right)+\int_{0}^{s\;\Delta t}f_{\mathrm{BW}}\left(\tau \right)\;d\tau =g_{\mathrm{BW}}\left(0\right)+\Delta t\sum_{i=1}^{s}f_{\mathrm{BW}}\left(i\;\Delta t\right)\\ & = & g_{\mathrm{BW}}\left(0\right)+f_{\mathrm{BW}}\left(0\right)\;s\;\Delta t+\frac{B_{0}}{2}\;N_{u0}\;{\left(s\;\Delta t\right)}^{2}+\\ & & +\sigma_{u}\;\Delta t^{5/2}\sum_{i=1}^{s}\sum_{j=1}^{s-i+1}j\;N_{\mathrm{ui}}+\sigma_{v}\;\Delta t^{3/2}\sum_{i=1}s\left(s-i+1\right)\;N_{\mathrm{vi}} \end{array}$$

(23) $$\begin{array}{ccc} E\left[g_{\mathrm{BW}}\left(s\;\Delta t\right)\right] & = & g_{\mathrm{BW}}\left(0\right)+f_{\mathrm{BW}}\left(0\right)\;\left(s\;\Delta t\right) \end{array}$$

(24) $$\begin{array}{ll} \mathrm{Var}\left[g_{\mathrm{BW}}\left(s\;\Delta t\right)\right] & =\frac{B_{0}^{2}}{4}\;{\left(s\;\Delta t\right)}^{4}+\sigma_{u}^{2}\;\Delta t^{5}\;\sum_{i=1}^{s}{\left(\sum_{j=1}^{s-i+1}j\right)}^{2}+\frac{\sigma_{v}^{2}}{6}\;\Delta t^{3}\;s\;\left(s+1\right)\;\left(2\;s+1\right)\\ & \approx \frac{B_{0}^{2}}{4}\;{\left(s\;\Delta t\right)}^{4}+\frac{\sigma_{u}^{2}}{20}\;{\left(s\;\Delta t\right)}^{5}+\frac{\sigma_{v}^{2}}{3}\;{\left(s\;\Delta t\right)}^{3} \end{array}$$

**Figure 6:**
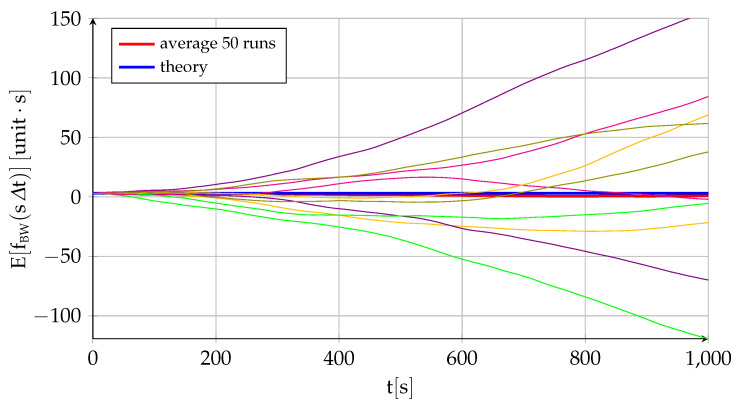
Propagation with time of second integral of sensor error mean.

**Figure 7:**
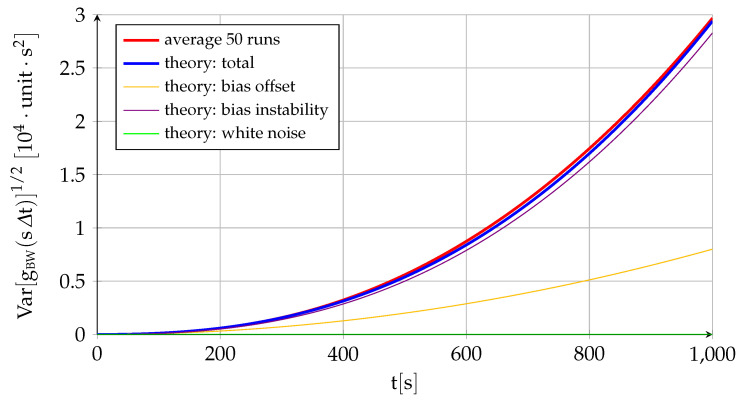
Propagation with time of second integral of sensor error standard deviation.

Figure 6 and Figure 7 show the same type of figures but applied to the second integral of the error ($g_{\mathrm{BW}}\left(0\right)=1.5$ has been employed in the experiment). In this case, the degradation of the results with time is even more intense to the point where the measurements are useless after a very short period of time. Unless corrected by the navigation system, this is equivalent to the error in position obtained by double integrating the output of the accelerometers.

Let us summarize the main points of the single-axis inertial sensor discrete error model developed in this section, which includes the influence of the bias and the system error but not that of the scale factor and cross-coupling errors included in the three-dimensional error model of Section 2.9. The error $e_{\mathrm{BW}}\left(s\;\Delta t\right)$, which applies to specific force for accelerometers and inertial angular velocity in the case of gyroscopes, depends on three factors: bias offset $B_{0}$, bias drift $\sigma_{u}$, and white noise $\sigma_{v}$. Its mean is always zero, but the error standard deviation grows with time ($\propto t^{1/2}$) due to the bias drift with constant contributions from the bias offset and the white noise. When integrating the error to obtain $f_{\mathrm{BW}}\left(s\;\Delta t\right)$, which is equivalent to ground velocity for accelerometers and attitude for gyroscopes, the initial speed error or initial attitude error $f_{\mathrm{BW}}\left(0\right)$ becomes the fourth contributor, and an important one indeed, as it becomes the mean of the first integral error at any time. The standard deviation, which measures the spread over the nonzero mean, increases very quickly with time because of the bias instability ($\propto \;t^{3/2}$), with contributions from the offset (∝t) and the white noise ($\propto \;t^{1/2}$). If integrating a second time to obtain $g_{\mathrm{BW}}\left(s\;\Delta t\right)$, or position in case of the accelerometer, the initial position error $g_{\mathrm{BW}}\left(0\right)$ turns into the fifth contributor. The expected value of the position error grows linearly with time due to the initial velocity error with an additional constant contribution from the initial position error, while the position standard deviation (measuring spread over a growing average value) grows extremely quick due mostly to the bias instability ($\propto \;t^{5/2}$) but also because of the bias offset ($\propto \;t^{2}$) and the white noise ($\propto \;t^{3/2}$). Table 4 shows the standard units of the different sources of error for both accelerometers and gyroscopes.

### 2.3. Obtainment of System Noise Values

This section focuses on the significance of system or white noise error $\sigma_{v}$ and how to obtain it from sensor specifications, which often refer to the integral of the output instead of the output itself. As the integral of white noise is a random walk process, the angle random walk of a gyroscope is equivalent to white noise in the angular rate output, while velocity random walk refers to the specific force white noise in accelerometers [37]. The discussion that follows applies to gyroscopes but is fully applicable to accelerometers if replacing the angular rate by specific force and attitude or angle by ground velocity.

Angle random walk, measured in ($\mathrm{rad}/s^{1/2}$), (${}^{\circ }/h^{1/2}$), or equivalent, describes the average deviation or error that occurs when the sensor output signal is integrated due to system noise exclusively, without considering other error sources such as bias or scale factor [41]. If integrating multiple times and obtaining a distribution of end points at a given final time sΔt, the standard deviation of this distribution, containing the final angles at the final time, scales linearly with the white noise level $\sigma_{v}$, the square root of the integration step size Δt, and the square root of the number of steps s, as noted by the last term of (21). This means that an angle random walk of 1 ${}^{\circ }/s^{1/2}$ translates into a standard deviation for the error of 1 ${}^{\circ }$ after 1 s, 10 ${}^{\circ }$ after 100 s, and $1000^{1/2}\approx 31.6$${}^{\circ }$ after 1000 s.

Manufacturers often provide this information as the power spectral density PSD of the white noise process in (${}^{\circ ,2}/h^{2}/\mathrm{Hz}$) or equivalent, where it is necessary to take its square root to obtain $\sigma_{v}$, or as the root PSD in (${}^{\circ }/h/{\mathrm{Hz}}^{1/2}$) that is equivalent to $\sigma_{v}$. Sometimes, it is even provided as the PSD of the random walk process, not the white noise, in units (${}^{\circ }/h$) or equivalent. It is then necessary to multiply this number by the square root of the sampling interval Δt or divide it by the square root of the sampling frequency to obtain the desired $\sigma_{v}$ value.

### 2.4. Obtainment of Bias Drift Values

This section describes the meaning of bias instability $\sigma_{u}$ (also known as bias stability or bias drift) and how to obtain it from sensor specifications. As in the previous section, the discussion is centered on gyroscopes, but it is fully applicable to accelerometers as well. Bias instability can be defined as the potential of the sensor error to stay within a certain range for a certain time [42]. A small number of manufacturers directly provide sensor output changes over time, which directly relates with the bias instability (also known as in-run bias variation, bias drift, or rate random walk) per the second term of (18). If provided with an angular rate change of x (${}^{\circ }/s$) (1σ) in t (s), then $\sigma_{u}$ can be obtained as follows [34,43]:

(25) $$\sigma_{u}=\frac{x}{t^{1/2}}$$

As the bias drift is responsible for the growth of sensor error with time (Figure 2 and Figure 3), manufacturers more commonly provide bias stability measurements that describe how the bias of a device may change over a specified period of time [35], typically around 100 s. Bias stability is usually specified as a 1σ value with units (${}^{\circ }/h$) or (${}^{\circ }/s$), which can be interpreted as follows according to (16)—(18). If the sensor error (or bias) is known at a given time t, then a 1σ bias stability of 0.01${}^{\circ }/h$ over 100 s means that the bias at time t+100 s is a random variable with the mean error at time t and standard deviation 0.01${}^{\circ }/h$, and expression (25) can be used to obtain $\sigma_{u}$. As the bias behaves as a random walk over time whose standard deviation grows proportionally to the square root of time, the bias stability is sometimes referred as a bias random walk.

In reality, bias fluctuations do not really behave as a random walk. If they did, the uncertainty in the bias of a device would grow without bound as the timespan increased, which is not the case. In practice, the bias is constrained to be within some range, and therefore, the random walk model is only a good approximation to the true process for short periods of time [35].

### 2.5. Platform, Accelerometers, and Gyroscopes Frames

The following sections make use of three different reference frames to describe the readings of accelerometers and gyroscopes:

- The *platform frame* $F_{P}$ is a Cartesian reference system with its origin located at the IMU reference point (Section 2.8) and its three axes $\left\{i_{1}^{P},\;i_{2}^{P},\;i_{3}^{P}\right\}$ forming a right-hand system that is loosely aligned with the aircraft body axes, so they point in the general directions of the aircraft fuselage (forward), aircraft wings (rightwards), and downward, respectively [2,31,32].
  A proper definition of the platform frame is indispensable for navigation, as the calibrated outputs of the accelerometers and gyroscopes are based on it (Section 2.6 and Section 2.7). The $F_{P}$ frame can be obtained from the body frame $F_{B}$ by a rotation best described by the Euler angles $\phi^{\mathrm{BP}}={\left[\psi^{P},\;\theta^{P},\;\xi^{P}\right]}^{T}$ (these Euler angles correspond to the 3–2–1 (yaw, pitch, roll) convention employed in aeronautics) followed by a translation $T_{\mathrm{BP}}^{B}$ (Section 2.8) from the aircraft center of mass to the IMU reference point.
- The *accelerometers frame* $F_{A}$ is a non-orthogonal reference system also centered at the IMU reference point [2,31,32]. The basis vectors $\left\{i_{1}^{A},\;i_{2}^{A},\;i_{3}^{A}\right\}$ are aligned with each of the three accelerometer’s sensing axes (each accelerometer hence only senses the specific force component parallel to its sensing axis) (Section 2.6), but they are not orthogonal among them due to manufacturing inaccuracies. This implies that the angles between the $F_{A}$ and $F_{P}$ axes are very small.
  It is always possible, with no loss of generality, to impose that $i_{1}^{P}$ coincides with $i_{1}^{A}$ and that $i_{2}^{P}$ is located in the plane defined by $i_{1}^{A}$ and $i_{2}^{A}$. If this is the case, $i_{1}^{A}⊥i_{2}^{P}$, $i_{1}^{A}⊥i_{3}^{P}$, and $i_{2}^{A}⊥i_{3}^{P}$, and the relative attitude between the $F_{P}$ and $F_{A}$ frames can be defined by three independent small rotations.
  − The $i_{2}^{A}$ axis can be obtained from $i_{2}^{P}$ by means of a small rotation $\alpha_{\mathrm{ACC},3}$ about $i_{3}^{P}$.
  − The $i_{3}^{A}$ axis can be obtained from $i_{3}^{P}$ by two small rotations: $\alpha_{\mathrm{ACC},1}$ about $i_{1}^{P}$ and $\alpha_{\mathrm{ACC},2}$ about $i_{2}^{P}$.
  Although the exact relationships can be obtained [31], and given that the angles are very small, it is possible to consider $\cos\alpha_{\mathrm{ACC},i}=1,\;\sin\alpha_{\mathrm{ACC},i}=\alpha_{\mathrm{ACC},i}$, and $\alpha_{\mathrm{ACC},i}\cdot \alpha_{\mathrm{ACC},j}=0\;\forall \;i,j\in \left\{1,\;2,\;3\right\},i\neq j$, resulting in the following transformations between free vectors viewed in the platform ($v^{P}$) and accelerometer ($v^{A}$) frames, respectively (As $F_{A}$ is not orthogonal, the transformation matrices are denoted with ⋆ to indicate that they are not proper rotation matrices):
  (26) $$\begin{array}{ccc} v^{P}=R_{\mathrm{PA}}^{⋆}\;v^{A} & = & \left[\begin{array}{ccc} 1 & 0 & 0\\ \alpha_{\mathrm{ACC},3} & 1 & 0\\ -\alpha_{\mathrm{ACC},2} & \alpha_{\mathrm{ACC},1} & 1 \end{array}\right]\;v^{A} \end{array}$$
  (27) $$\begin{array}{ccc} v^{A}=R_{\mathrm{AP}}^{⋆}\;v^{P} & = & \left[\begin{array}{ccc} 1 & 0 & 0\\ -\alpha_{\mathrm{ACC},3} & 1 & 0\\ \alpha_{\mathrm{ACC},2} & -\alpha_{\mathrm{ACC},1} & 1 \end{array}\right]\;v^{P} \end{array}$$
- The *gyroscopes frame* $F_{Y}$ is similar to the accelerometers frame $F_{A}$ defined above, but it is aligned with the gyroscopes’ sensing axes instead of those of the accelerometers [2,31,32]. It is also a non-orthogonal reference system centered at the IMU reference point, but no simplifications can be made about the relative orientation of its axes $\left\{i_{1}^{Y},\;i_{2}^{Y},\;i_{3}^{Y}\right\}$ with respect to those of $F_{P}$, so their relative attitude is defined by six small rotations $\alpha_{\mathrm{GYR},\mathrm{ij}}\;\forall \;i,j\in \left\{1,\;2,\;3\right\},i\neq j$, where $\alpha_{\mathrm{GYR},\mathrm{ij}}$ is the rotation of $i_{i}^{Y}$ about $i_{j}^{P}$.
  An approach similar to that employed with accelerometers leads to the following transformations between free vectors viewed in the platform ($v^{P}$) and gyroscope ($v^{Y}$) frames:
  (28) $$\begin{array}{ccc} v^{P}=R_{\mathrm{PY}}^{⋆}\;v^{Y} & = & \left[\begin{array}{ccc} 1 & -\alpha_{\mathrm{GYR},23} & \alpha_{\mathrm{GYR},32}\\ \alpha_{\mathrm{GYR},13} & 1 & -\alpha_{\mathrm{GYR},31}\\ -\alpha_{\mathrm{GYR},12} & \alpha_{\mathrm{GYR},21} & 1 \end{array}\right]\;v^{Y} \end{array}$$
  (29) $$\begin{array}{ccc} v^{Y}=R_{\mathrm{YP}}^{⋆}\;v^{P} & = & \left[\begin{array}{ccc} 1 & \alpha_{\mathrm{GYR},23} & -\alpha_{\mathrm{GYR},32}\\ -\alpha_{\mathrm{GYR},13} & 1 & \alpha_{\mathrm{GYR},31}\\ \alpha_{\mathrm{GYR},12} & -\alpha_{\mathrm{GYR},21} & 1 \end{array}\right]\;v^{P} \end{array}$$

### 2.6. Accelerometer Triad Sensor Error Model

An IMU is equipped with an accelerometer triad composed by three individual accelerometers, each of which measures the projection of the specific force over its sensing axis as described in Section 2.2 while incurring in an error $e_{\mathrm{BW},\mathrm{ACC}}$ that can be modeled as a combination of bias offset, bias drift, and white noise (16). The three accelerometers can be considered infinitesimally small and located at the IMU *reference point*, which is defined as the intersection between the sensing axes of the three accelerometers. As the accelerometer frame $F_{A}$ is centered at the IMU reference point and its three non-orthogonal axes coincide with the accelerometers’ sensing axes, (30) joins together the measurements of the three individual accelerometers:

(30) $${\tilde{f}}_{\mathrm{IA}}^{A}=S_{\mathrm{ACC}}\;\left(f_{\mathrm{IA}}^{A}+e_{\mathrm{BW},\mathrm{ACC}}^{A}\right)$$

where $f_{\mathrm{IA}}^{A}$ is the specific force viewed in the accelerometer frame $F_{A}$, ${\tilde{f}}_{\mathrm{IA}}^{A}$ represents its measurement also viewed in $F_{A}$, $e_{\mathrm{BW},\mathrm{ACC}}^{A}$ is the error introduced by each accelerometer (16), and $S_{\mathrm{ACC}}$ is a square diagonal matrix containing the scale factor errors $\left\{s_{\mathrm{ACC},1},\;s_{\mathrm{ACC},2},\;s_{\mathrm{ACC},3}\right\}$ for each accelerometer (Section 2.1). It is however preferred to obtain an expression in which the specific forces are viewed in the orthogonal platform frame $F_{P}$ instead of the accelerometer frame $F_{A}$. As both share the same origin,

(31) $${\tilde{f}}_{\mathrm{IP}}^{P}=R_{\mathrm{PA}}^{⋆}\;S_{\mathrm{ACC}}\;\left(R_{\mathrm{AP}}^{⋆}\;f_{\mathrm{IP}}^{P}+e_{\mathrm{BW},\mathrm{ACC}}^{A}\right)$$

where $R_{\mathrm{PA}}^{⋆}$ and $R_{\mathrm{AP}}^{⋆}$, defined by (26) and (27), contain the cross-coupling errors $\left\{\alpha_{\mathrm{ACC},1},\;\alpha_{\mathrm{ACC},2},\;\alpha_{\mathrm{ACC},3}\right\}$ generated by the misalignment of the accelerometer sensing axes. The scale factor and cross-coupling errors contain fixed and temperature-dependent error contributions (refer to Section 2.1) that can be modeled as normal random variables:

(32) $$\begin{array}{ccc} s_{\mathrm{ACC},i} & = & N\left(1,\;s_{\mathrm{ACC}}^{2}\right)\;\;\forall \;i\in \left\{1,\;2,\;3\right\} \end{array}$$

(33) $$\begin{array}{ccc} \alpha_{\mathrm{ACC},i} & = & N\left(0,\;\alpha_{\mathrm{ACC}}^{2}\right)\;\forall \;i\in \left\{1,\;2,\;3\right\} \end{array}$$

where $s_{\mathrm{ACC}}$ and $\alpha_{\mathrm{ACC}}$ can be obtained from the sensor specifications. Equation (31) can be transformed to make it more useful by defining the accelerometer scale and cross-coupling error matrix $M_{\mathrm{ACC}}$:

(34) $$\begin{array}{ccc} M_{\mathrm{ACC}} & = & R_{\mathrm{PA}}^{⋆}\;S_{\mathrm{ACC}}\;R_{\mathrm{AP}}^{⋆}=\left[\begin{array}{ccc} m_{\mathrm{ACC},11} & 0 & 0\\ m_{\mathrm{ACC},21} & m_{\mathrm{ACC},22} & 0\\ m_{\mathrm{ACC},31} & m_{\mathrm{ACC},32} & m_{\mathrm{ACC},33} \end{array}\right]\\ & \approx & \left[\begin{array}{ccc} s_{\mathrm{ACC},1} & 0 & 0\\ \alpha_{\mathrm{ACC},3}\;\left(s_{\mathrm{ACC},1}-s_{\mathrm{ACC},2}\right) & s_{\mathrm{ACC},2} & 0\\ \alpha_{\mathrm{ACC},2}\;\left(s_{\mathrm{ACC},3}-s_{\mathrm{ACC},1}\right) & \alpha_{\mathrm{ACC},1}\;\left(s_{\mathrm{ACC},2}-s_{\mathrm{ACC},3}\right) & s_{\mathrm{ACC},3} \end{array}\right] \end{array}$$

Considering that the scale and cross-coupling errors are uncorrelated and very small, and taking into account the expressions for the mean and variance of the sum and product of two random variables [44], the different components $m_{\mathrm{ACC},\mathrm{ij}}$ of $M_{\mathrm{ACC}}$ can be obtained as follows $\forall \;i,\;j\in \left\{1,\;2,\;3\right\}$:

(35) $$\begin{array}{ccc} m_{\mathrm{ACC},\mathrm{ij}} & = & N\left(1,\;s_{\mathrm{ACC}}^{2}\right)\;i=j \end{array}$$

(36) $$\begin{array}{ccc} m_{\mathrm{ACC},\mathrm{ij}} & = & N\left(0,\;{\left[\sqrt{2}\;\alpha_{\mathrm{ACC}}\;s_{\mathrm{ACC}}\right]}^{2}\right)=N\left(0,m_{\mathrm{ACC}}^{2}\right)\;i>j \end{array}$$

(37) $$\begin{array}{ccc} m_{\mathrm{ACC},\mathrm{ij}} & = & 0\;i<j \end{array}$$

Let us also define the accelerometer error transformation matrix $N_{\mathrm{ACC}}$ as

(38) $$N_{\mathrm{ACC}}=R_{\mathrm{PA}}^{⋆}S_{\mathrm{ACC}}=\left[\begin{array}{ccc} n_{\mathrm{ACC},11} & 0 & 0\\ n_{\mathrm{ACC},21} & n_{\mathrm{ACC},22} & 0\\ n_{\mathrm{ACC},31} & n_{\mathrm{ACC},32} & n_{\mathrm{ACC},33} \end{array}\right]=\left[\begin{array}{ccc} s_{\mathrm{ACC},1} & 0 & 0\\ \alpha_{\mathrm{ACC},3}\;s_{\mathrm{ACC},1} & s_{\mathrm{ACC},2} & 0\\ -\alpha_{\mathrm{ACC},2}\;s_{\mathrm{ACC},1} & \alpha_{\mathrm{ACC},1}\;s_{\mathrm{ACC},2} & s_{\mathrm{ACC},3} \end{array}\right]$$

A process similar to that employed above leads to:

(39) $$\begin{array}{ccc} n_{\mathrm{ACC},\mathrm{ij}} & = & N\left(1,\;s_{\mathrm{ACC}}^{2}\right)\;i=j \end{array}$$

(40) $$\begin{array}{ccc} n_{\mathrm{ACC},\mathrm{ij}} & = & N\left(0,\;\alpha_{\mathrm{ACC}}^{2}\left(1+s_{\mathrm{ACC}}^{2}\right)\right)\approx N\left(0,\alpha_{\mathrm{ACC}}^{2}\right)\;i>j \end{array}$$

(41) $$\begin{array}{ccc} n_{\mathrm{ACC},\mathrm{ij}} & = & 0\;i<j \end{array}$$

Taking into account the expressions for the mean and variance of the sum and product of two random variables [44], and knowing that the cross-coupling errors are very small $\left(1+\alpha_{\mathrm{ACC}}^{2}\approx 1\right)$, it can be proven that the bias and white noise errors viewed in the platform frame $F_{P}$ respond to a expression similar to (16):

(42) $$\begin{array}{ccc} e_{\mathrm{BW},\mathrm{ACC}}^{P} & = & e_{\mathrm{BW},\mathrm{ACC}}^{P}\left(s\;\Delta t_{\mathrm{SENSED}}\right)=e_{\mathrm{BW},\mathrm{ACC}}^{P}\left(s\;\Delta t\right)\\ & = & N_{\mathrm{ACC}}\;e_{\mathrm{BW},\mathrm{ACC}}^{A}=B_{0\mathrm{ACC}}\;N_{u0,\mathrm{ACC}}+\sigma_{u\mathrm{ACC}}\;\Delta t^{1/2}\;\sum_{i=1}^{s}N_{\mathrm{ui},\mathrm{ACC}}+\frac{\sigma_{v\mathrm{ACC}}}{\Delta t^{1/2}}\;N_{\mathrm{vs},\mathrm{ACC}} \end{array}$$

where each $N_{u,\mathrm{ACC}}$ and $N_{v,\mathrm{ACC}}$ is a random vector composed by three independent standard normal random variables $N\left(0,\;1\right)$. Note that as the bias drift is mostly a warm-up process that stabilizes itself after a few minutes of operation, the random walk within (42) is not allowed to vary freely but is restricted to within a band of width $\pm \;100\;\sigma_{u\mathrm{ACC}}\;\Delta t^{1/2}$. The final model for the accelerometer measurements viewed in $F_{P}$ results in

(43) $${\tilde{f}}_{\mathrm{IP}}^{P}=M_{\mathrm{ACC}}\;f_{\mathrm{IP}}^{P}+e_{\mathrm{BW},\mathrm{ACC}}^{P}$$

where $M_{\mathrm{ACC}}$ is described in (34) through (37) and $e_{\mathrm{BW},\mathrm{ACC}}^{P}$ is provided by (42). This model relies on inputs for the bias offset $B_{0\mathrm{ACC}}$, bias drift $\sigma_{u\mathrm{ACC}}$, white noise $\sigma_{v\mathrm{ACC}}$, scale factor error $s_{\mathrm{ACC}}$, and cross-coupling error $m_{\mathrm{ACC}}$. Section 6.2 provides an example on how to obtain these values from the data sheet provided by the accelerometer manufacturer.

### 2.7. Gyroscopes Triad Sensor Error Model

The IMU is also equipped with a triad of gyroscopes, each of which measures the projection of the inertial angular velocity over its sensing axis as described in Section 2.2. The obtainment of the gyroscope triad model is fully analogous to that of the accelerometers in the previous section, with the added difficulty that the transformation between the gyroscope frame $F_{Y}$ and platform frame $F_{P}$ relies on six small angles instead of three. The starting point hence is:

(44) $${\tilde{\omega }}_{\mathrm{IP}}^{P}=R_{\mathrm{PY}}^{⋆}\;S_{\mathrm{GYR}}\;\left(R_{\mathrm{YP}}^{⋆}\;\omega_{\mathrm{IP}}^{P}+e_{\mathrm{BW},\mathrm{GYR}}^{Y}\right)$$

where $\omega_{\mathrm{IP}}^{P}$ is the inertial angular velocity viewed in the platform frame $F_{P}$, ${\tilde{\omega }}_{\mathrm{IP}}^{P}$ represents its measurement also viewed in $F_{P}$, $e_{\mathrm{BW},\mathrm{GYR}}^{Y}$ is the error introduced by each gyroscope (16), $S_{\mathrm{GYR}}$ is a square diagonal matrix containing the scale factor errors $\left\{s_{\mathrm{GYR},1},\;s_{\mathrm{GYR},2},\;s_{\mathrm{GYR},2}\right\}$, and $R_{\mathrm{PY}}^{⋆}$ and $R_{\mathrm{YP}}^{⋆}$, defined by (28) and (29), contain the cross-coupling errors $\alpha_{\mathrm{GYR},12},\;\alpha_{\mathrm{GYR},21},\;\alpha_{\mathrm{GYR},13},\;\alpha_{\mathrm{GYR},31},\;\alpha_{\mathrm{GYR},23},\;\alpha_{\mathrm{GYR},32}$ generated by the misalignment of the gyroscope sensing axes.

Operating in the same way as in Section 2.6 leads to:

(45) $$\begin{array}{ll} e_{\mathrm{BW},\mathrm{GYR}}^{P} & =e_{\mathrm{BW},\mathrm{GYR}}^{P}\left(s\;\Delta t_{\mathrm{SENSED}}\right)=e_{\mathrm{BW},\mathrm{GYR}}^{P}\left(s\;\Delta t\right)\\ & =B_{0\mathrm{GYR}}\;N_{u0,\mathrm{GYR}}+\sigma_{u\mathrm{GYR}}\;\Delta t^{1/2}\;\sum_{i=1}^{s}N_{\mathrm{ui},\mathrm{GYR}}+\frac{\sigma_{v\mathrm{GYR}}}{\Delta t^{1/2}}\;N_{\mathrm{vs},\mathrm{GYR}} \end{array}$$

(46) $$\begin{array}{ccc} {\tilde{\omega }}_{\mathrm{IP}}^{P} & = & M_{\mathrm{GYR}}\;\omega_{\mathrm{IP}}^{P}+e_{\mathrm{BW},\mathrm{GYR}}^{P} \end{array}$$

where each $N_{\mathrm{ui},\mathrm{GYR}}$ and $N_{v,\mathrm{GYR}}$ is a random vector composed by three independent standard normal random variables $N\left(0,\;1\right)$. As in the case of the accelerometers, the random walk within (45) representing the bias drift is not allowed to vary freely but is restricted to within a band of width $\pm \;100\;\sigma_{u\mathrm{GYR}}\;\Delta t^{1/2}$. This model relies on inputs for the bias offset $B_{0\mathrm{GYR}}$, bias drift $\sigma_{u\mathrm{GYR}}$, white noise $\sigma_{v\mathrm{GYR}}$, scale factor error $s_{\mathrm{GYR}}$, and cross-coupling error $m_{\mathrm{GYR}}$, which can be obtained from the gyroscope specifications. An example of this process is included in Section 6.2. The gyroscope scale and cross-coupling error matrix $M_{\mathrm{GYR}}$ responds to:

(47) $$\begin{array}{ccc} M_{\mathrm{GYR}} & = & R_{\mathrm{PY}}^{⋆}\;S_{\mathrm{GYR}}\;R_{\mathrm{YP}}^{⋆}=\left[\begin{array}{ccc} m_{\mathrm{GYR},11} & m_{\mathrm{GYR},12} & m_{\mathrm{GYR},13}\\ m_{\mathrm{GYR},21} & m_{\mathrm{GYR},22} & m_{\mathrm{GYR},23}\\ m_{\mathrm{GYR},31} & m_{\mathrm{GYR},32} & m_{\mathrm{GYR},33} \end{array}\right]\\ & \approx & \left[\begin{array}{ccc} s_{\mathrm{GYR},1} & \alpha_{\mathrm{GYR},23}\;\left(s_{\mathrm{GYR},1}-s_{\mathrm{GYR},2}\right) & \alpha_{\mathrm{GYR},32}\;\left(s_{\mathrm{GYR},3}-s_{\mathrm{GYR},1}\right)\\ \alpha_{\mathrm{GYR},13}\;\left(s_{\mathrm{GYR},1}-s_{\mathrm{GYR},2}\right) & s_{\mathrm{GYR},2} & \alpha_{\mathrm{GYR},31}\;\left(s_{\mathrm{GYR},2}-s_{\mathrm{GYR},3}\right)\\ \alpha_{\mathrm{GYR},12}\;\left(s_{\mathrm{GYR},3}-s_{\mathrm{GYR},1}\right) & \alpha_{\mathrm{GYR},21}\;\left(s_{\mathrm{GYR},2}-s_{\mathrm{GYR},3}\right) & s_{\mathrm{GYR},3} \end{array}\right] \end{array}$$

(48) $$\begin{array}{ccc} m_{\mathrm{GYR},\mathrm{ij}} & = & N\left(1,\;s_{\mathrm{GYR}}^{2}\right)\;i=j \end{array}$$

(49) $$\begin{array}{ccc} m_{\mathrm{GYR},\mathrm{ij}} & = & N\left(0,\;2\;\alpha_{\mathrm{GYR}}^{2}\;s_{\mathrm{GYR}}^{2}\right)=N\left(0,\;m_{\mathrm{GYR}}^{2}\right)\;i\neq j \end{array}$$

### 2.8. Mounting of Inertial Sensors

Equations (43) and (46) contain the relationships between the specific force $f_{\mathrm{IP}}^{P}$ and inertial angular velocity $\omega_{\mathrm{IP}}^{P}$ and their measurements $\left({\tilde{f}}_{\mathrm{IP}}^{P},\;{\tilde{\omega }}_{\mathrm{IP}}^{P}\right)$ when evaluated and viewed in the platform frame $F_{P}$. However, from the point of view of the navigation system, both magnitudes need to be evaluated and viewed in the body frame $F_{B}$ instead of $F_{P}$. These equations thus need to be modified so they relate $f_{\mathrm{IB}}^{B}$ with ${\tilde{f}}_{\mathrm{IB}}^{B}$ as well as $\omega_{\mathrm{IB}}^{B}$ with ${\tilde{\omega }}_{\mathrm{IB}}^{B}$, respectively, as described in Section 2.9 below. To do that, it is necessary to define the relative pose (position plus attitude) between the $F_{P}$ and $F_{B}$ frames, and to distinguish between the true position $T_{\mathrm{BP}}^{B}$ and attitude $\phi^{\mathrm{BP}}$, and their estimations by the IMU processor (${\hat{T}}_{\mathrm{BP}}^{B}$ and ${\hat{\phi }}^{\mathrm{BP}}$). Note that the IMU, represented by the platform frame $F_{P}$, should be mounted in the aircraft as close as possible to the center of gravity (this reduces errors, as described in Section 2.9), and it is loosely aligned with the aircraft body axes.

To increase the realism, this article assumes that the real displacement $T_{\mathrm{BP}}^{B}$ between the two frames is deterministic, while the relative rotation $\phi^{\mathrm{BP}}={\left[\psi^{P},\;\theta^{P},\;\xi^{P}\right]}^{T}$ is stochastic. In this way, each simulation run exhibits a slightly different IMU platform attitude with respect to the aircraft body:

- As the IMU reference point is fixed with respect to the structure but the aircraft center of mass slowly moves as the fuel load diminishes, it is possible to establish a linear model that provides the displacement between the origins of both frames according to the aircraft mass (the aircraft masses $m_{\mathrm{full}}$ and $m_{\mathrm{empty}}$) when the fuel tank is fully loaded or empty as inputs, as are the displacements between the IMU reference point and the aircraft center of mass $T_{\mathrm{BP},\mathrm{full}}^{B}$ and $T_{\mathrm{BP},\mathrm{empty}}^{B}$.:
  (50) $$T_{\mathrm{BP}}^{B}=f\left(m\right)=T_{\mathrm{BP},\mathrm{full}}^{B}+\frac{m_{\mathrm{full}}-m}{m_{\mathrm{full}}-m_{\mathrm{empty}}}\;\left(T_{\mathrm{BP},\mathrm{empty}}^{B}-T_{\mathrm{BP},\mathrm{full}}^{B}\right)$$
- The platform Euler angles respond to the stochastic model provided by (51), in which each specific Euler angle is obtained as the product of the user-selected standard deviations ($\sigma_{\psi^{P}}$, $\sigma_{\theta^{P}}$, $\sigma_{\xi^{P}}$) by a single realization of a standard normal random variable $N\left(0,\;1\right)$ ($N_{\psi^{P}}$, $N_{\theta^{P}}$, and $N_{\xi^{P}}$).
  (51) $$\phi^{\mathrm{BP}}={\left[\sigma_{\psi^{P}}\;N_{\psi^{P}},\;\sigma_{\theta^{P}}\;N_{\theta^{P}},\;\sigma_{\xi^{P}}N_{\xi^{P}}\right]}^{T}$$

Once the real pose between the $F_{P}$ and $F_{B}$ frames is established, it is necessary to specify its estimation employed by the IMU processor in the comprehensive model introduced in Section 2.9, which is discussed in Section 5.2. Stochastic models are employed for both the translation ${\hat{T}}_{\mathrm{BP}}^{B}$ and rotation ${\hat{\phi }}^{\mathrm{BP}}$, changing their values from one execution to the next:

(52) $$\begin{array}{ccc} {\hat{T}}_{\mathrm{BP}}^{B} & = & T_{\mathrm{BP}}^{B}+{\left[\sigma_{{\hat{T}}_{\mathrm{BP}}^{B}}\;N_{{\hat{T}}_{\mathrm{BP},1}^{P}},\;\sigma_{{\hat{T}}_{\mathrm{BP}}^{B}}\;N_{{\hat{T}}_{\mathrm{BP},2}^{P}},\;\sigma_{{\hat{T}}_{\mathrm{BP}}^{B}}N_{{\hat{T}}_{\mathrm{BP},3}^{P}}\right]}^{T} \end{array}$$

(53) $$\begin{array}{ccc} {\hat{\phi }}^{\mathrm{BP}} & = & \phi^{\mathrm{BP}}+{\left[\sigma_{{\hat{\phi }}^{\mathrm{BP}}}\;N_{{\hat{\psi }}^{P}},\;\sigma_{{\hat{\phi }}^{\mathrm{BP}}}\;N_{{\hat{\theta }}^{P}},\;\sigma_{{\hat{\phi }}^{\mathrm{BP}}}N_{{\hat{\xi }}^{P}}\right]}^{T} \end{array}$$

As in the previous case, the model relies on two user-selected standard deviations ($\sigma_{{\hat{T}}_{\mathrm{BP}}^{B}}$ and $\sigma_{{\hat{\phi }}^{\mathrm{BP}}}$), as well as six realizations of a standard normal random variable $N\left(0,\;1\right)$, which are denoted as $N_{{\hat{\psi }}^{P}}$, $N_{{\hat{\theta }}^{P}}$, $N_{{\hat{\xi }}^{P}}$, $N_{{\hat{T}}_{\mathrm{BP},1}^{P}}$, $N_{{\hat{T}}_{\mathrm{BP},2}^{P}}$, and $N_{{\hat{T}}_{\mathrm{BP},3}^{P}}$. Section 6.2 suggests values for the five required settings ($\sigma_{\psi^{P}},\;\sigma_{\theta^{P}},\;\sigma_{\xi^{P}},\;\sigma_{{\hat{T}}_{\mathrm{BP}}^{B}},\;\sigma_{{\hat{\phi }}^{\mathrm{BP}}}$), although they can be adjusted by the user.

$T_{\mathrm{BP}}$ can be considered quasi-stationary as it slowly varies based on the aircraft mass, and the relative position of their axes $\phi^{\mathrm{BP}}$ remains constant because the IMU is rigidly attached to the aircraft structure. Although Euler angles have been employed in this section, from this point on, it is more practical to employ the rotation matrix $R_{\mathrm{BP}}$ to represent the rotation between two different frames [45]. The time derivatives of $T_{\mathrm{BP}}$ and $R_{\mathrm{BP}}$ are hence zero:

(54) $${\dot{T}}_{\mathrm{BP}}={\dot{R}}_{\mathrm{BP}}=0\;⟶\;v_{\mathrm{BP}}=a_{\mathrm{BP}}=\omega_{\mathrm{BP}}=\alpha_{\mathrm{BP}}=0$$

### 2.9. Comprehensive Inertial Sensor Error Model

Two considerations are required to establish the measurement equations for the inertial sensors viewed in the body frame $F_{B}$. First, let us apply the composition rules of Appendix A.6 considering $F_{I}$ as $F_{0}$, $F_{B}$ as $F_{1}$, and $F_{P}$ as $F_{2}$, which results in:

(55) $$\begin{array}{ccc} \omega_{\mathrm{IP}} & = & \omega_{\mathrm{IB}} \end{array}$$

(56) $$\begin{array}{ccc} \alpha_{\mathrm{IP}} & = & \alpha_{\mathrm{IB}} \end{array}$$

(57) $$\begin{array}{ccc} v_{\mathrm{IP}} & = & v_{\mathrm{IB}}+{\hat{\omega }}_{\mathrm{IB}}\;T_{\mathrm{BP}} \end{array}$$

(58) $$\begin{array}{ccc} a_{\mathrm{IP}} & = & a_{\mathrm{IB}}+{\hat{\alpha }}_{\mathrm{IB}}\;T_{\mathrm{BP}}+{\hat{\omega }}_{\mathrm{IB}}\;{\hat{\omega }}_{\mathrm{IB}}\;T_{\mathrm{BP}} \end{array}$$

Second, it is also necessary to consider that as ${\hat{R}}_{\mathrm{BP}}$ is a rotation matrix in which all rows and columns are unitary vectors, the projection of the $F_{P}$ frame bias and white noise errors $e_{\mathrm{BW},\mathrm{ACC}}^{P}$ and $e_{\mathrm{BW},\mathrm{GYR}}^{P}$ onto the $F_{B}$ frame does not change their stochastic properties:

(59) $$e_{\mathrm{BW},\mathrm{ACC}}^{B}\left(s\Delta t\right)={\hat{R}}_{\mathrm{BP}}e_{\mathrm{BW},\mathrm{ACC}}^{P}=B_{0\mathrm{ACC}}N_{u0,\mathrm{ACC}}+\sigma_{u\mathrm{ACC}}\Delta t^{1/2}\;\sum_{i=1}^{s}N_{\mathrm{ui},\mathrm{ACC}}+\frac{\sigma_{v\mathrm{ACC}}}{\Delta t^{1/2}}N_{\mathrm{vs},\mathrm{ACC}}$$

(60) $$e_{\mathrm{BW},\mathrm{GYR}}^{B}\left(s\Delta t\right)={\hat{R}}_{\mathrm{BP}}e_{\mathrm{BW},\mathrm{GYR}}^{P}=B_{0\mathrm{GYR}}N_{u0,\mathrm{GYR}}+\sigma_{u\mathrm{GYR}}\Delta t^{1/2}\;\sum_{i=1}^{s}N_{\mathrm{ui},\mathrm{GYR}}+\frac{\sigma_{v\mathrm{GYR}}}{\Delta t^{1/2}}N_{\mathrm{vs},\mathrm{GYR}}$$

As the inertial angular velocity does not change when evaluated in the $F_{B}$ and $F_{P}$ frames (55), its measurement in the body frame can be derived from (46) by first projecting it from $F_{B}$ to $F_{P}$ based on the real rotation matrix $R_{\mathrm{BP}}$ and then projecting back the measurement into $F_{B}$ based on the estimated rotation matrix ${\hat{R}}_{\mathrm{BP}}$. The bias and white noise error is also projected according to (60):

(61) $${\tilde{\omega }}_{\mathrm{IB}}^{B}={\hat{R}}_{\mathrm{BP}}\;M_{\mathrm{GYR}}\;{R_{\mathrm{BP}}}^{T}\;\omega_{\mathrm{IB}}^{B}+e_{\mathrm{BW},\mathrm{GYR}}^{B}$$

The expression for the specific force measurement is significantly more complex because the back and forth transformations of the specific force between the $F_{B}$ and $F_{P}$ frames need to consider the influence of the lever arm $T_{\mathrm{BP}}$, as indicated in (58). The additional terms introduce errors in the measurements, so as indicated in Section 2.8, it is desirable to locate the IMU as close as possible to the aircraft center of mass.

(62) $${\tilde{f}}_{\mathrm{IB}}^{B}={\hat{R}}_{\mathrm{BP}}\;M_{\mathrm{ACC}}\;{R_{\mathrm{BP}}}^{T}\;\left(f_{\mathrm{IB}}^{B}+{\hat{\alpha }}_{\mathrm{IB}}^{B}\;T_{\mathrm{BP}}^{B}+{\hat{\omega }}_{\mathrm{IB}}^{B}\;{\hat{\omega }}_{\mathrm{IB}}^{B}\;T_{\mathrm{BP}}^{B}\right)-{\hat{\hat{\alpha }}}_{\mathrm{IB}}^{B}\;{\hat{T}}_{\mathrm{BP}}^{B}-{\hat{\hat{\omega }}}_{\mathrm{IB}}^{B}\;{\hat{\hat{\omega }}}_{\mathrm{IB}}^{B}\;{\hat{T}}_{\mathrm{BP}}^{B}+e_{\mathrm{BW},\mathrm{ACC}}^{B}$$

Note that this expression cannot be directly evaluated as the estimated values for the inertial angular velocity and acceleration (${\hat{\omega }}_{\mathrm{IB}}^{B},\;{\hat{\alpha }}_{\mathrm{IB}}^{B}$) are unknown by the IMU until obtained by the navigation filter. The IMU can however rely on the gyroscope readings, directly replacing ${\hat{\omega }}_{\mathrm{IB}}^{B}$ with ${\tilde{\omega }}_{\mathrm{IB}}^{B}$ and computing ${\tilde{\alpha }}_{\mathrm{IB}}^{B}$ based on the difference between the present and previous ${\tilde{\omega }}_{\mathrm{IB}}^{B}$ readings, resulting in:

(63) $${\tilde{f}}_{\mathrm{IB}}^{B}={\hat{R}}_{\mathrm{BP}}\;M_{\mathrm{ACC}}\;{R_{\mathrm{BP}}}^{T}\;\left(f_{\mathrm{IB}}^{B}+{\hat{\alpha }}_{\mathrm{IB}}^{B}\;T_{\mathrm{BP}}^{B}+{\hat{\omega }}_{\mathrm{IB}}^{B}\;{\hat{\omega }}_{\mathrm{IB}}^{B}\;T_{\mathrm{BP}}^{B}\right)-{\hat{\tilde{\alpha }}}_{\mathrm{IB}}^{B}\;{\hat{T}}_{\mathrm{BP}}^{B}-{\hat{\tilde{\omega }}}_{\mathrm{IB}}^{B}\;{\hat{\tilde{\omega }}}_{\mathrm{IB}}^{B}\;{\hat{T}}_{\mathrm{BP}}^{B}+e_{\mathrm{BW},\mathrm{ACC}}^{B}$$

Table 5 lists the error sources contained in the comprehensive inertial sensor error model represented by (61), (63). The first two columns list the different error sources, while the third column specifies their origin according to the criterion established in the first paragraph of Section 2.1. The section where each error is described appears on the fourth column, which is followed by the seeds (refer to Section 6 for the meaning of the terms $\Upsilon_{i,A}$ and $\Upsilon_{j,F}$) employed to ensure the results variability for different aircraft ($\Upsilon_{i,A}$) as well as different flights ($\Upsilon_{j,F}$).

Note that all the required error sources (2nd column) need to be specified by the user. As an example, Section 6.2 suggests values appropriate for a low SWaP aircraft. It is worth pointing out that all errors are modeled as stochastic variables or processes (with the exception of the $T_{\mathrm{BP}}$ displacement between the body and platform frames, which is deterministic), as expressions (61), (63) rely on the errors provided by (59), (60), the scale and cross-coupling matrices given by (34), (47), and the transformations given by (50), (51), (52), (53).

In the case of the accelerometer triad, the stochastic nature of the fixed and run-to-run error contributions to the model relies on three realizations of normal distributions for the bias offset, three for the scale factor errors, three for the cross-coupling errors, and nine for the mounting errors, while the in-run error contributions require three realizations each for the bias drift and system noise at every discrete sensor measurement. The gyroscope triad is similar but requires six realizations to model the cross-coupling errors instead of three while using the same six realizations as the accelerometer triad to model the true and estimated rotation between the $F_{B}$ and $F_{P}$ frames.

Expressions (61), (63) can be rewritten to show the measurements as functions of the full errors ($E_{\mathrm{ACC}},\;E_{\mathrm{GYR}}$), which represent all the errors introduced by the inertial sensors with the exception of white noise.

(64) $$\begin{array}{ccc} {\tilde{f}}_{\mathrm{IB}}^{B}\left(s\;\Delta t\right) & = & f_{\mathrm{IB}}^{B}\left(s\;\Delta t\right)+E_{\mathrm{ACC}}\left(s\;\Delta t\right)+\frac{\sigma_{v\mathrm{ACC}}}{\Delta t^{1/2}}\;N_{\mathrm{vs},\mathrm{ACC}} \end{array}$$

(65) $$\begin{array}{ccc} {\tilde{\omega }}_{\mathrm{IB}}^{B}\left(s\;\Delta t\right) & = & \omega_{\mathrm{IB}}^{B}\left(s\;\Delta t\right)+E_{\mathrm{GYR}}\left(s\;\Delta t\right)+\frac{\sigma_{v\mathrm{GYR}}}{\Delta t^{1/2}}\;N_{\mathrm{vs},\mathrm{GYR}} \end{array}$$

## 3. Non-Inertial Sensors

This section describes the different non-inertial sensors usually installed onboard a fixed wind autonomous aircraft, such as a triad of *magnetometers* to measure the Earth’s magnetic field, a *GNSS receiver* that provides absolute position and velocity measurements, and the *air data system*, which in addition to the pressure altitude and temperature also provides a measurement of the airspeed and the airflow angles.

### 3.1. Magnetometers

Magnetometers measure magnetic field intensity along a given direction and are very useful for estimating the aircraft heading. Although other types exist, magnetoinductive and magnetoresistive sensors are generally employed for navigation due to their accuracy and small size [4,30]. As with the inertial sensors, three orthogonal magnetometers are usually employed in a strapdown configuration to measure the magnetic field with respect to the body frame $F_{B}$.

Unfortunately, magnetometers do not only measure the Earth’s magnetic field B but also that generated by the aircraft permanent magnets and electrical equipment (known as hard iron magnetism) as well as the magnetic field disturbances generated by the aircraft ferrous materials (soft iron magnetism). For that reason, the magnetometers should be placed in a location inside the aircraft that minimizes these errors. On the positive side, magnetometers do not exhibit the bias instability present in inertial sensors, and the error of an individual sensor can be properly modeled by the combination of bias offset and white noise. A triad of magnetometers capable of measuring the magnetic field in three directions adds the same scale factor (nonlinearity) and cross-coupling (misalignment) errors as those present in the inertial sensors, together with the transformation between the magnetic axes and the body ones.

Modeling the behavior of a triad of magnetometers is simpler but less precise than that of inertial sensors, as the effect of the fixed hard iron magnetism is indistinguishable from that of the run-to-run bias offset, while the fixed effect of soft iron magnetism is indistinguishable from that of the scale factor and cross-coupling error matrix. This has several consequences. First of all is that magnetometers cannot be calibrated at the laboratory before being mounted in the aircraft as in the case of inertial sensors (Section 5.1) but are instead calibrated once attached to the aircraft by a process known as swinging (Section 5.3), which is less precise, as the aircraft attitude during swinging cannot be determined with so much accuracy as it would be in a laboratory setting. Second is that defining a magnetic platform frame to then transform the results into body axes serves no purpose, as the magnetometer readings are only valid, this is, contain the effects of hard and soft iron magnetism, once they are attached to the aircraft, and then they can be directly measured in body axes. Third is that percentage-wise, the errors induced by the magnetometers are bigger than those of the inertial sensors. The implemented model is the following:

(66) $$\begin{array}{ccc} {\tilde{B}}^{B} & = & B_{\mathrm{HI},\mathrm{MAG}}+B_{0,\mathrm{MAG}}+M_{\mathrm{MAG}}\;R_{\mathrm{BN}}\;B^{N}+e_{W,\mathrm{MAG}}^{B} \end{array}$$

(67) $$\begin{array}{ccc} {\tilde{B}}^{B}\left(s\;\Delta t\right) & = & B_{\mathrm{HI},\mathrm{MAG}}\;N_{\mathrm{HI},\mathrm{MAG}}+B_{0,\mathrm{MAG}}\;N_{u0,\mathrm{MAG}}+M_{\mathrm{MAG}}\;R_{\mathrm{BN}}\;B^{N}+\frac{\sigma_{v,\mathrm{MAG}}}{\Delta t^{1/2}}\;N_{\mathrm{vs},\mathrm{MAG}} \end{array}$$

where ${\tilde{B}}^{B}$ is the measurement viewed in $F_{B}$, $B_{\mathrm{HI},\mathrm{MAG}}$ is the fixed hard iron magnetism, $B_{0,\mathrm{MAG}}$ is the run-to-run bias offset, $M_{\mathrm{MAG}}$ is a fixed matrix combining the effects of soft iron magnetism with the scale factor and cross-coupling errors, and $B^{N}$ is the real magnetic field including local anomalies. $N_{\mathrm{HI},\mathrm{MAG}}$, $N_{u0,\mathrm{MAG}}$ and $N_{\mathrm{vs},\mathrm{MAG}}$ are uncorrelated normal vectors of size three each composed of three uncorrelated standard normal random variables $N\left(0,\;1\right)$. The soft iron, scale factor and cross-coupling matrix $M_{\mathrm{MAG}}$ does not vary with time and is computed as follows:

(68) $$M_{\mathrm{MAG}}=\left[\begin{array}{ccc} s_{\mathrm{MAG}} & m_{\mathrm{MAG}} & m_{\mathrm{MAG}}\\ m_{\mathrm{MAG}} & s_{\mathrm{MAG}} & m_{\mathrm{MAG}}\\ m_{\mathrm{MAG}} & m_{\mathrm{MAG}} & s_{\mathrm{MAG}} \end{array}\right]\circ N_{m,\mathrm{MAG}}$$

In this expression, $N_{m,\mathrm{MAG}}$ contains nine outputs of a standard normal random variable $N\left(0,\;1\right)$, and the symbol ∘ represents the Hadamart or element-wise matrix product.

Table 6 lists the error sources contained in the magnetometer model in the same format as the previous section, noting that soft iron magnetism is included in both the scale factor and cross-coupling errors. Note that all the required error sources ($B_{\mathrm{HI},\mathrm{MAG}},\;B_{0,\mathrm{MAG}},\;\sigma_{v,\mathrm{MAG}},\;s_{\mathrm{MAG}},m_{\mathrm{MAG}}$) need to be specified by the user. As an example, Section 6.2 suggests values appropriate for a low SWaP aircraft. The stochastic nature of the fixed and run-to-run error contributions to the magnetometer model relies on three realizations of normal distributions for the hard iron magnetism, three for the bias offset, three for the scale factor errors, and six for the cross-coupling errors, while the in-run error contributions require three realizations for system noise at every discrete sensor measurement.

Expression (67) can be rewritten to show the measurements as functions of the magnetometer full error $E_{\mathrm{MAG}}$, which represents all the errors introduced by the magnetometers with the exception of white noise:

(69) $${\tilde{B}}^{B}\left(s\;\Delta t\right)=B^{B}\left(s\;\Delta t\right)+E_{\mathrm{MAG}}\left(s\;\Delta t\right)+\frac{\sigma_{v,\mathrm{MAG}}}{\Delta t^{1/2}}\;N_{\mathrm{vs},\mathrm{MAG}}$$

### 3.2. Global Navigation Satellite System Receiver

A GNSS receiver enables the determination of the aircraft position and absolute velocity based on signals obtained from various constellations of satellites, such as GPS, GLONASS, and Galileo. The position is obtained by triangulation based on the accurate satellite position and time contained within each signal. Instead of derivating the position with respect to time, which introduces noise, GNSS receivers obtain the vehicle absolute velocity by measuring the Doppler shift between the constant satellite frequencies and those measured by the receiver.

It is important to note that because of the heavy processing required to fix a position based on the satellite signals, GNSS receivers are not capable of working at the high frequencies characteristic of inertial and air data sensors, so $\Delta t_{\mathrm{GNSS}}$ is usually a multiple of $\Delta t_{\mathrm{SENSED}}$. The position error of a GNSS receiver can be modeled as the sum of a zero mean white noise process plus slow varying ionospheric effects [33] modeled as the sum of the bias offset plus a random walk. This random walk is modeled with a frequency of 1/60Hz ($\Delta t_{\mathrm{ION}}=60\;s$) and linearly interpolated in between. The ground velocity error is modeled exclusively with a white noise process.

(70) $$\begin{array}{ccc} e_{\mathrm{GNSS},\mathrm{POS}}\left(g\;\Delta t_{\mathrm{GNSS}}\right) & = & {\tilde{x}}_{\mathrm{GDT}}-x_{\mathrm{GDT}}=\sigma_{\mathrm{GNSS},\mathrm{POS}}\;N_{g,\mathrm{GNSS},\mathrm{POS}}+e_{\mathrm{GNSS},\mathrm{ION}}\left(g\;\Delta t_{\mathrm{GNSS}}\right) \end{array}$$

(71) $$e_{\mathrm{GNSS},\mathrm{VEL}}\left(g\;\Delta t_{\mathrm{GNSS}}\right)={\tilde{v}}^{N}-v^{N}=\sigma_{\mathrm{GNSS},\mathrm{VEL}}\;N_{g,\mathrm{GNSS},\mathrm{VEL}}$$

(72) $$\begin{array}{ll} e_{\mathrm{GNSS},\mathrm{ION}}\left(g\;\Delta t_{\mathrm{GNSS}}\right) & =e_{\mathrm{GNSS},\mathrm{ION}}\left(i\;\Delta t_{\mathrm{ION}}\right)+\\ & \frac{r}{f_{\mathrm{ION}}}\left(e_{\mathrm{GNSS},\mathrm{ION}}\left(\left(i+1\right)\Delta t_{\mathrm{ION}}\right)-e_{\mathrm{GNSS},\mathrm{ION}}\left(i\;\Delta t_{\mathrm{ION}}\right)\right) \end{array}$$

(73) $$\begin{array}{ccc} g & = & f_{\mathrm{ION}}\cdot i+r\;\;\;\;\;\;\;\;\;0\leq r<f_{\mathrm{ION}} \end{array}$$

(74) $$\begin{array}{ccc} e_{\mathrm{GNSS},\mathrm{ION}}\left(i\;\Delta t_{\mathrm{ION}}\right) & = & B_{0,\mathrm{GNSS},\mathrm{ION}}\;N_{u0,\mathrm{GNSS},\mathrm{ION}}+\sigma_{\mathrm{GNSS},\mathrm{ION}}\;\sum_{j=1}^{i}N_{j,\mathrm{GNSS},\mathrm{ION}} \end{array}$$

(75) $$\begin{array}{ccc} f_{\mathrm{ION}} & = & \Delta t_{\mathrm{ION}}/\Delta t_{\mathrm{GNSS}}=60 \end{array}$$

where $\sigma_{\mathrm{GNSS},\mathrm{POS}}$, $\sigma_{\mathrm{GNSS},\mathrm{ION}}$, $B_{0,\mathrm{GNSS},\mathrm{ION}}$, and $\sigma_{\mathrm{GNSS},\mathrm{VEL}}$ are user-supplied inputs (Section 6.2 contains an example on how to fill up these values), and $N_{g,\mathrm{GNSS},\mathrm{POS}}$, $N_{g,\mathrm{GNSS},\mathrm{VEL}}$, $N_{u0,\mathrm{GNSS},\mathrm{ION}}$, and $N_{j,\mathrm{GNSS},\mathrm{ION}}$ are uncorrelated normal vectors of size three, each composed of three uncorrelated standard normal random variables $N\left(0,\;1\right)$. In addition, note that as both g and $f_{\mathrm{ION}}$ are integers, the quotient remainder theorem guarantees that there exist unique integers i and r that comply with (73) [46].

Table 7 lists the error sources contained in the GNSS receiver model in the same format as previous sections. Note that all errors are modeled as stochastic variables or processes. Three realizations of a normal distribution are required for the run-to-run error contributions, while the in-run error contributions require three realizations each for position and velocity at every discrete sensor measurement, plus an extra three when corresponding for the ionospheric error.

### 3.3. Air Data System

The mission of the air data system is to measure the aircraft pressure altitude $H_{P}$ [47,48] by means of the atmospheric pressure p, the outside air temperature T, the airspeed $v_{\mathrm{TAS}}$, and the angles of attack α and sideslip β that provide the orientation of the aircraft structure with respect to the airflow.

A barometer or static pressure sensor, generally part of the Pitot tube as explained below [30], measures atmospheric pressure, which can be directly translated into pressure altitude [47,48]. Air data systems are also equipped with a thermometer to measure the external air temperature T. The implemented models, where OSP stands for outside static pressure and OAT means outside air temperature, include contributions from both bias offsets ($B_{0\mathrm{OSP}},\;B_{0\mathrm{OAT}}$) and random noises ($\sigma_{\mathrm{OSP}},\;\sigma_{\mathrm{OAT}}$):

(76) $$\begin{array}{ccc} e_{\mathrm{OSP}}\left(s\;\Delta t_{\mathrm{SENSED}}\right) & = & e_{\mathrm{OSP}}\left(s\;\Delta t\right)=\tilde{p}\left(s\;\Delta t\right)-p\left(s\;\Delta t\right)=B_{0\mathrm{OSP}}\;N_{0,\mathrm{OSP}}+\sigma_{\mathrm{OSP}}\;N_{s,\mathrm{OSP}} \end{array}$$

(77) $$\begin{array}{ccc} e_{\mathrm{OAT}}\left(s\;\Delta t_{\mathrm{SENSED}}\right) & = & e_{\mathrm{OAT}}\left(s\;\Delta t\right)=\tilde{T}\left(s\;\Delta t\right)-T\left(s\;\Delta t\right)=B_{0\mathrm{OAT}}\;N_{0,\mathrm{OAT}}+\sigma_{\mathrm{OAT}}\;N_{s,\mathrm{OAT}} \end{array}$$

where $N_{0,\mathrm{OSP}}$, $N_{s,\mathrm{OSP}}$, $N_{0,\mathrm{OAT}}$, and $N_{s,\mathrm{OAT}}$ are uncorrelated standard normal random variables $N\left(0,\;1\right)$.

A *Pitot probe* is a tube with no outlet pointing directly into the undisturbed air stream, where the values of the air variables (temperature, pressure, and density) at its dead end resemble the total or stagnation variables of the atmosphere prior to its deceleration inside the Pitot [49]. Measuring the air flow total pressure $p_{t}$ at the tube dead end enables the estimation of the aircraft airspeed $v_{\mathrm{TAS}}$.

The air data system is also capable of measuring the direction of the air stream with respect to the aircraft, which is represented by the angles of attack α and sideslip β. To do so, it can be equipped with two air vanes that align themselves with the unperturbed air stream or with a more complex multi-hole Pitot probe. In all three cases, the errors can also be modeled by a combination of bias offsets ($B_{0\mathrm{TAS}},\;B_{0\mathrm{AOA}},\;B_{0\mathrm{AOS}}$) and random noises ($\sigma_{\mathrm{TAS}},\;\sigma_{\mathrm{AOA}},\;\sigma_{\mathrm{AOS}}$):

(78) $$e_{\mathrm{TAS}}\left(s\;\Delta t_{\mathrm{SENSED}}\right)=e_{\mathrm{TAS}}\left(s\;\Delta t\right)={\tilde{v}}_{\mathrm{TAS}}\left(s\;\Delta t\right)-v_{\mathrm{TAS}}\left(s\;\Delta t\right)=B_{0\mathrm{TAS}}\;N_{0,\mathrm{TAS}}+\sigma_{\mathrm{TAS}}\;N_{s,\mathrm{TAS}}$$

(79) $$e_{\mathrm{AOA}}\left(s\;\Delta t_{\mathrm{SENSED}}\right)=e_{\mathrm{AOA}}\left(s\;\Delta t\right)=\tilde{\alpha }\left(s\;\Delta t\right)-\alpha \left(s\;\Delta t\right)=B_{0\mathrm{AOA}}\;N_{0,\mathrm{AOA}}+\sigma_{\mathrm{AOA}}\;N_{s,\mathrm{AOA}}$$

(80) $$e_{\mathrm{AOS}}\left(s\;\Delta t_{\mathrm{SENSED}}\right)=e_{\mathrm{AOS}}\left(s\;\Delta t\right)=\tilde{\beta }\left(s\;\Delta t\right)-\beta \left(s\;\Delta t\right)=B_{0\mathrm{AOS}}\;N_{0,\mathrm{AOS}}+\sigma_{\mathrm{AOS}}\;N_{s,\mathrm{AOS}}$$

where $N_{0,\mathrm{TAS}}$, $N_{s,\mathrm{TAS}}$, $N_{0,\mathrm{AOA}}$, $N_{s,\mathrm{AOA}}$, $N_{0,\mathrm{AOS}}$, and $N_{s,\mathrm{AOS}}$ are uncorrelated standard normal random variables $N\left(0,\;1\right)$. Table 8 lists the error sources contained in the air data sensor model represented by (76), (77), (78), (79), and (80) in the same format as previous tables. Note that all errors are modeled as stochastic variables or processes. The stochastic nature of the run-to-run error contributions to the models relies on five realizations of normal distributions for the bias offsets, while the in-run error contributions require five realizations for the system noises at every discrete sensor measurement.

## 4. Camera

Image generation is a power and data-intensive process that cannot work at the high frequencies characteristic of inertial and air data sensors, so $\Delta t_{\mathrm{IMG}}$ is usually significantly higher than $\Delta t_{\mathrm{SENSED}}$ but not as much as $\Delta t_{\mathrm{GNSS}}$. The camera is considered rigidly attached to the aircraft structure, and it is assumed that the shutter speed is sufficiently high so that all images are equally sharp, and that the image generation process is instantaneous. In addition, the camera ISO setting remains constant during the flight, and all generated images are noise free. The model also assumes that the visible spectrum radiation reaching all patches of the Earth’s surface remains constant, and the terrain is considered Lambertian [50], so its appearance at any given time does not vary with the viewing direction. The combined use of these assumptions implies that a given terrain object is represented with the same luminosity in all images, even as its relative pose (position and attitude) with respect to the camera varies. Geometrically, a perspective projection or pinhole camera model [50] is employed, which in addition is perfectly calibrated and hence shows no distortion. Table 9 lists the required configuration parameters, which shall be provided by the user.

### 4.1. Mounting of Camera

The digital camera can be located anywhere on the aircraft structure as long as its view of the terrain is unobstructed by other platform elements. It is desirable that the lever arm or distance between the camera optical center and the aircraft center of mass is as small as possible to reduce the negative effects of any camera alignment error. With respect to its orientation, the camera should be facing down to show a balanced view of the ground during level flight, but minor deviations are not problematic.

As in the case of the IMU platform, the model considers that the camera location is deterministic but its orientation is stochastic. The expressions below are hence analogous to those employed in Section 2.8, where each specific camera Euler angle is obtained as the product of the standard deviations ($\sigma_{\psi^{C}}$, $\sigma_{\theta^{C}}$, $\sigma_{\xi^{C}}$) by a single realization of a standard normal random variable $N\left(0,\;1\right)$ ($N_{\psi^{C}}$, $N_{\theta^{C}}$, and $N_{\xi^{C}}$):

(81) $$\begin{array}{ccc} T_{\mathrm{BC}}^{B} & = & f\left(m\right)=T_{\mathrm{BC},\mathrm{full}}^{B}+\frac{m_{\mathrm{full}}-m}{m_{\mathrm{full}}-m_{\mathrm{empty}}}\;\left(T_{\mathrm{BC},\mathrm{empty}}^{B}-T_{\mathrm{BC},\mathrm{full}}^{B}\right) \end{array}$$

(82) $$\begin{array}{ccc} \phi_{\mathrm{BC}} & = & {\left[90{\;}^{\circ }+\sigma_{\psi^{C}}\;N_{\psi^{C}},\;\sigma_{\theta^{C}}\;N_{\theta^{C}},\;\sigma_{\xi^{C}}N_{\xi^{C}}\right]}^{T} \end{array}$$

In addition to the true translation and rotation between the $F_{B}$ and $F_{C}$ frames given by the previous equations, the model also requires the accuracy with which they are known to the navigation system. The determination of the camera position ${\hat{T}}_{\mathrm{BC}}^{B}$ and rotation ${\hat{\phi }}^{\mathrm{BC}}={\left[{\hat{\psi }}^{C},\;{\hat{\theta }}^{C},\;{\hat{\xi }}^{C}\right]}^{T}$ is discussed in Section 5.4. As in previous cases, stochastic models are considered for both the translation ${\hat{T}}_{\mathrm{BC}}^{B}$ and rotation ${\hat{\phi }}^{\mathrm{BC}}$, changing their values from one run to another:

(83) $$\begin{array}{ccc} {\hat{T}}_{\mathrm{BC}}^{B} & = & T_{\mathrm{BC}}^{B}+{\left[\sigma_{{\hat{T}}_{\mathrm{BC}}^{B}}\;N_{{\hat{T}}_{\mathrm{BC},1}^{B}},\;\sigma_{{\hat{T}}_{\mathrm{BC}}^{B}}\;N_{{\hat{T}}_{\mathrm{BC},2}^{B}},\;\sigma_{{\hat{T}}_{\mathrm{BC}}^{B}}N_{{\hat{T}}_{\mathrm{BC},3}^{B}}\right]}^{T} \end{array}$$

(84) $$\begin{array}{ccc} {\hat{\phi }}^{\mathrm{BC}} & = & \phi_{\mathrm{BC}}+{\left[\sigma_{{\hat{\phi }}^{\mathrm{BC}}}\;N_{{\hat{\psi }}^{C}},\;\sigma_{{\hat{\phi }}^{\mathrm{BC}}}\;N_{{\hat{\theta }}^{C}},\;\sigma_{{\hat{\phi }}^{\mathrm{BC}}}N_{{\hat{\xi }}^{C}}\right]}^{T} \end{array}$$

where $N_{{\hat{\psi }}^{C}}$, $N_{{\hat{\theta }}^{C}}$, $N_{{\hat{\xi }}^{C}}$, $N_{{\hat{T}}_{\mathrm{BC},1}^{B}}$, $N_{{\hat{T}}_{\mathrm{BC},2}^{B}}$, and $N_{{\hat{T}}_{\mathrm{BC},3}^{B}}$ are six realizations of a standard normal random variable $N\left(0,\;1\right)$. Section 6.2 provides an example of the standard deviations required to fill up the model, although they can be adjusted by the user.

The translation $T_{\mathrm{BC}}^{B}$ between the origins of the $F_{B}$ and $F_{C}$ frames can be considered quasi-stationary, as it slowly varies based on the aircraft mass (81), and the relative position of their axes $\phi_{\mathrm{BC}}$ remains constant because the camera is rigidly attached to the aircraft structure (82).

### 4.2. Earth Viewer

**Figure 8:**
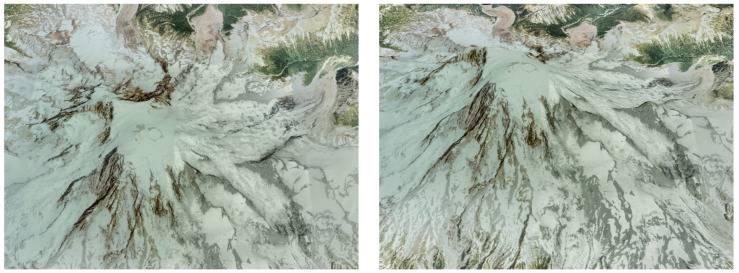
Example of Earth Viewer images.

The camera model differs from all other sensor models described in this article in that it does not return a sensed variable $\tilde{x}$ consisting of its real value x plus a sensor error E but instead generates a digital image simulating what a real camera would record based on the aircraft position and attitude as given by the actual or real state $x=x_{\mathrm{TRUTH}}$. When provided with the camera pose with respect to the Earth at equally time-spaced intervals, the available model implementation [6] is capable of generating images that resemble the view of the Earth’s surface that the camera would record if located at that particular pose. To do so, it relies on three software libraries:

- OpenSceneGraph [51] is an open-source high-performance 3D graphics toolkit written in C++ and OpenGL, used by application developers in fields such as visual simulation, games, virtual reality, scientific visualization and modeling. The library enables the representation of objects in a scene by means of a graph data structure, which allows grouping objects that share some properties to automatically manage rendering properties such as the level of detail necessary to faithfully draw the scene but without considering the unnecessary detail that slows down the graphics hardware drawing the scene.
- osgEarth [52] is a dynamic and scalable 3D Earth surface rendering toolkit that relies on OpenSceneGraph, and it is based on publicly available *orthoimages* of the area flown by the aircraft. Orthoimages consist of aerial or satellite imagery geometrically corrected such that the scale is uniform; they can be used to measure true distances as they are accurate representations of the Earth surface, having been adjusted for topographic relief, lens distortion, and camera tilt. When coupled with a *terrain elevation model*, osgEarth is capable of generating realistic images based on the camera position as well as its yaw and pitch, but it does not accept the camera roll (in other words, the osgEarth images are always aligned with the horizon).
- Earth Viewer is a modification to osgEarth implemented by the authors, so it is also capable of accepting the bank angle of the camera with respect to the NED axes. Earth Viewer is capable of generating realistic Earth images as long as the camera height over the terrain is significantly higher than the vertical relief present in the image. As an example, Figure 8 shows two different views of a volcano in which the dome of the mountain, having very steep slopes, is properly rendered.

## 5. Calibration Procedures

This section describes various calibration processes required for the determination of the fixed and run-to-run error contributions to the accelerometers, gyroscopes, magnetometers, and onboard camera. These procedures only need to be executed once and do not need to be repeated unless the sensors are replaced or their position inside the aircraft is modified (in addition, the swinging process of Section 5.3 needs to be performed every time new equipment is installed inside the aircraft, as this may modify the hard and soft iron magnetism and hence the magnetometer readings).

The calibration procedures include the laboratory calibration of the accelerometers and gyroscopes described in Section 5.1, the determination of the pose between the platform and body frames explained in Section 5.2, the magnetometer calibration or swinging described in Section 5.3, and the determination of the pose between the camera and body frames explained in Section 5.4. Their main objective is the determination of the fixed contributions to the sensor error models (refer to Section 2.1 for the different types of sensor error contributions, including fixed, run-to-run, and in-run), that is, the scale factor and cross-coupling errors of both inertial sensors and magnetometers (${\hat{M}}_{\mathrm{ACC}},\;{\hat{M}}_{\mathrm{GYR}},\;{\hat{M}}_{\mathrm{MAG}}$) (note that ${\hat{M}}_{\mathrm{MAG}}$ also includes the soft iron magnetism), the magnetometers hard iron magnetism ${\hat{B}}_{\mathrm{HI},\mathrm{MAG}}$, the body to platform transformation (${\hat{T}}_{\mathrm{BP}}^{B},\;{\hat{\phi }}^{\mathrm{BP}}$), and the body-to-camera transformation (${\hat{T}}_{\mathrm{BC}}^{B},\;{\hat{\phi }}^{\mathrm{BC}}$). These procedures also provide estimations for the run-to-run error contributions (${\hat{B}}_{0\mathrm{ACC}},\;{\hat{B}}_{0\mathrm{GYR}},\;{\hat{B}}_{0,\mathrm{MAG}}$), but these need to be discarded, as they change every time the aircraft systems are switched on.

### 5.1. Inertial Sensors Calibration

*Calibration* is the process of comparing instrument outputs with known references to determine coefficients that force the outputs to agree with the references over a range of output values [31]. The IMU inertial sensors need to be calibrated to eliminate the fixed errors originated from manufacturing and also to determine their temperature sensitivity [32]. The calibration process requires significant material and time resources, but it greatly reduces the measurement errors. While high-grade IMUs are always factory calibrated, low-cost ones generally are not, so it is necessary to calibrate the IMU at the laboratory before mounting it on the aircraft [4].

The calibration process is executed at a location where the position $x_{\mathrm{GDT}}$ and the gravity (gravity includes both gravitation and centrifugal accelerations) vector $g_{c}$ have been previously determined with great precision [31]. It relies on a three-axis table, which enables rotating the IMU with known angular velocities into a set of predetermined precisely controlled orientations [32,53]. Accelerometer and gyroscope measurements are then compared to reference values (gravity for the accelerometers, torquing rate plus Earth angular velocity for the gyroscopes) and the differences employed to generate corrections [31].

During calibration, the amount of time that the IMU is maintained stationary at each attitude, as well as the time required to rotate it between two positions, are trade-offs based on two opposing influences. On one side, longer periods of time are preferred as the negative influence of the system noise in the measurements tends to even out over time, while on the other, shorter times imply smaller variations of the bias drift over the measurement interval.

It is worth noting that as the calibration is performed before the IMU is installed on the aircraft, it relies on the platform frame $F_{P}$ and the models contained in Section 2.6 and Section 2.7. Although it is possible to use a calibration strategy based on selecting platform orientations that isolate sensor input onto a single axis (for example, gravity will only be sensed by the accelerometer that is placed vertically with respect to the Earth’s surface) to then apply least squares techniques, in real life, it is better to employ state estimation techniques (the estimation filter not only relies on known gravity and angular velocity but also the fact that the IMU is stationary and hence its velocity is zero) to obtain estimates of the inertial sensor’s scale factors, cross-coupling errors, and bias offsets [4,32]. The process is repeated at different temperatures so the IMU processor can later apply the correction based on the IMU sensor temperature [4].

The twenty-one coefficients estimated in the calibration process are listed in Table 10. Once the coefficients have been estimated, they can be introduced into the IMU processor so it automatically performs the corrections contained in (85) and (86):

(85) $$\begin{array}{ccc} {\tilde{\tilde{f}}}_{\mathrm{IP}}^{P} & = & {\hat{M}}_{\mathrm{ACC}}^{-1}\;{\tilde{f}}_{\mathrm{IP}}^{P}-{\hat{B}}_{0\mathrm{ACC}} \end{array}$$

(86) $$\begin{array}{ccc} {\tilde{\tilde{\omega }}}_{\mathrm{IP}}^{P} & = & {\hat{M}}_{\mathrm{GYR}}^{-1}\;{\tilde{\omega }}_{\mathrm{IP}}^{P}-{\hat{B}}_{0\mathrm{GYR}} \end{array}$$

This article assumes that the bias offset is exclusively a run-to-run source of error that varies every time the IMU is switched on, so the ${\hat{B}}_{0\mathrm{ACC}}$ and ${\hat{B}}_{0\mathrm{GYR}}$ coefficients obtained by calibration are discarded, as they have no relation to the offsets that occur during flight. Modeling the results obtained by the calibration process implies reducing the scale factor and cross-couplings errors found on the inertial sensors specifications by an arbitrary amount that can be specified by the user. To summarize, instead of applying (85), (86) to the measurements obtained by (61), (63), the model directly employs (61), (63) with reduced $M_{\mathrm{GYR}}$ and $M_{\mathrm{ACC}}$ values.

### 5.2. Determination of the Platform Frame Pose

The true relative pose between the body and platform frames ($F_{B}$, $F_{P}$), given by $T_{\mathrm{BP}}^{B}$ and $\phi^{\mathrm{BP}}$, as well as their estimated values ${\hat{T}}_{\mathrm{BP}}^{B}$ and ${\hat{\phi }}^{\mathrm{BP}}$, play a key role in the readings generated by the inertial sensors, as explained in Section 2.9.

Considering that the position of the aircraft center of mass is known (in both full and empty tank configurations), the true displacement $T_{\mathrm{BP}}^{B}$ can be determined with near exactitude based on the IMU attachment point to the aircraft, resulting in very low $\sigma_{{\hat{T}}_{\mathrm{BP}}^{B}}$ values to be employed for the estimation of ${\hat{T}}_{\mathrm{BP}}^{B}$ in (52).

With regard to the attitude $\phi^{\mathrm{BP}}$, after mounting the IMU platform so two of its axes are approximately aligned with the forward and down directions of an approximate aircraft plane of symmetry (with no particular need for accuracy), it is possible to estimate the angular deviation $\phi^{\mathrm{BP}}$ by means of self-alignment [4], resulting in small $\sigma_{{\hat{\phi }}^{\mathrm{BP}}}$ values when estimating ${\hat{\phi }}^{\mathrm{BP}}$ in (53).

### 5.3. Swinging or Magnetometer Calibration

Magnetometer calibration is inherently more complex than that of the inertial sensors, as it must be performed with the sensors already mounted on the aircraft, as otherwise, it would not capture the fixed contributions of the hard iron and soft iron magnetisms (Section 3.1). The calibration process, known as *swinging*, relies on obtaining magnetometer readings while the aircraft is positioned at different attitudes that encompass a wide array of heading, pitch, and roll values [4], and it is executed at a location where the magnetic field is precisely known.

The accuracy of the results is very dependent of the precision with which the different aircraft attitudes can be determined during swinging. This can be done with self-alignment procedures [4] or with the use of expensive static instruments. In any case, attitude accuracy is always going to be inferior to that obtained with a three-axis table during inertial sensor calibration. Once the magnetic field readings are obtained, they are compared to the real magnetic field values, and expression (67) is employed with least squares techniques to obtain estimations of the bias (sum of hard iron magnetism $B_{\mathrm{HI},\mathrm{MAG}}$ and offset $B_{0,\mathrm{MAG}}$), and the scale factor and cross-coupling matrix $M_{\mathrm{MAG}}$, which also includes the soft iron magnetism. The process can be repeated several times to isolate the influence of hard iron magnetism (a fixed effect that does not change) from the offset, which is a run-to-run error source that changes every time the magnetometers are turned on.

The fifteen coefficients estimated in the swinging process are listed in Table 11. Once the coefficients have been estimated, they can be introduced into the processor so it automatically performs the corrections shown in (87):

(87) $${\tilde{\tilde{B}}}^{B}={\hat{M}}_{\mathrm{MAG}}^{-1}\;{\tilde{B}}^{B}-{\hat{B}}_{\mathrm{HI},\mathrm{MAG}}-{\hat{B}}_{0,\mathrm{MAG}}$$

This articles assumes that the bias offset $B_{0,\mathrm{MAG}}$ is exclusively a run-to-run source of error that varies every time the magnetometer is switched on, so bias offset coefficients obtained by swinging are discarded, as they have no relation to the offsets that occur during flight. Modeling the results obtained by swinging implies reducing the hard iron bias $B_{\mathrm{HI},\mathrm{MAG}}$ and scale factor and cross-coupling errors $M_{\mathrm{MAG}}$ found on the sensor’s specifications by an arbitrary amount that can be specified by the user. To summarize, instead of applying (87) to the measurements obtained by (67), the model directly employs (67) with reduced $B_{\mathrm{HI},\mathrm{MAG}}$ and $M_{\mathrm{MAG}}$ values.

### 5.4. Determination of the Camera Frame Pose

The images generated by the onboard camera, and simulated by means of the Earth Viewer application introduced in Section 4.2, do not only depend on the relative pose between the body and the Earth but also on that of the camera with respect to the aircraft structure, which is represented by the rotation $\phi_{\mathrm{BC}}$ and displacement $T_{\mathrm{BC}}^{B}$ generated when mounting the camera, as described in Section 4.1. Visual navigation algorithms, however, rely on the navigation system best estimate of this pose, this is, ${\hat{\phi }}^{\mathrm{BC}}$ and ${\hat{T}}_{\mathrm{BC}}^{B}$, which need to be estimated once the already calibrated camera has been mounted on the aircraft. The two-phase process requires a chess board such as that employed for camera calibration [50,54].

The first phase uses an optimization procedure quite similar to that used in calibration to determine the relative pose between the camera frame $F_{C}$ and the one rigidly attached to the chessboard. Instead of using the location of each chess box corner in different images, this process relies on a single photo and imposes that all chess boxes are square and have the same size, which is enough to obtain a solution up to an unknown scale. The size of the chess boxes provides the scale required to unambiguously solve the identification problem with high precision.

The second step is to obtain the pose between the chessboard and body frames. This is a straightforward geometric optimization problem that relies on distance measurements between chessboard points and aircraft structure points whose coordinates in the $F_{B}$ frame are known. The resulting accuracy depends on the accuracy with which these distances can be measured, so special equipment may be required given the importance of the final estimations for the success of the visual navigation algorithms.

Overall, this is a robust and accurate process if properly executed, which results in the user-selectable $\sigma_{{\hat{T}}_{\mathrm{BC}}^{B}}$$\sigma_{{\hat{\phi }}^{\mathrm{BC}}}$ values employed for the stochastic estimation of ${\hat{T}}_{\mathrm{BC}}^{B}$ and ${\hat{\phi }}^{\mathrm{BC}}$ in each run by means of (83) and (84).

## 6. Discussion: Realism, Stochastic Properties and Customizable Inputs

This article provides models of the various sensors usually installed onboard a fixed wing autonomous aircraft, and it includes a ready-to-use open source C++ implementation [6] so researchers can quickly generate realistic, stochastic (pseudo-random), and customizable time-stamped series of the outputs of the different sensors, including images of the Earth’s surface that closely resemble what a real camera would record from the same positions and attitudes.

- In terms of realism, the detailed descriptions of Section 2, Section 3 and Section 4 show that in addition to the usually present white noise and random walk contributions, the models also include key error sources such as scale factors and cross-coupling effects, which are challenging for navigation systems, since these often include some type of linearization, and inputs that should theoretically be restricted to a single axis (caused by maneuvering or turbulence) in fact result in outputs to all three sensor axes. Supplied with a series of time-varying aircraft positions and attitudes, the Earth Viewer application generates detailed distortion-free images, resembling what a real onboard camera would record if flying the same trajectory.
  In addition, and only for the case of the camera and inertial sensors, the models do not only consider the relative pose (position and attitude) between the platform (IMU) and camera frames (in which the information is recorded) and the body frame (into which they are converted for output), as well as their slow variations with time as a consequence of fuel load changes, but also take into account the inaccuracies in the aircraft processors’ knowledge of these relative poses.
- With respect to randomness, the random nature of the outputs generated by the various sensors is reflected in the extensive use of stochastic processes and distributions within the models. Section 6.1 below explains how all sensor errors are derived from two input seeds (one identifying the aircraft or fixed errors, and another specifying the flight, or run-to-run and in-run error contributions). This facilitates the use of the models within Monte Carlo simulations while also enabling the same set of outputs to be reproduced if so desired.
- Regarding the customization, the realism in the models relies on multiple input parameters that need to be provided by the end user. Most of them can be found in the data sheets provided by the manufacturers (usually with different names and conventions, which are explained in previous sections), others depend on the methods employed for the mounting of the sensors onboard the aircraft, and some of them can be improved by means of the calibration procedures described in Section 5. Section 6.2 describes the process followed to obtain the parameters for the case of a small low SWaP aircraft, which constitutes the default configuration for the model’s C++ implementation [6].

### 6.1. Stochastic Models and the Use of Input Seeds

As explained in the corresponding sections, the outputs of the different sensor models depend on the input seeds listed in Table 5, Table 6, Table 7 and Table 8 and grouped together in Table 12 for convenience.

The following steps describe how these seeds are obtained in the available C++ implementation of the models [6]:

1. Initialize a discrete uniform distribution with any seed (any value is valid, so 1 was employed by the authors), which produces random integers where each possible value has an equal likelihood of being produced. Call this distribution a number of times equal or higher than twice the maximum number of runs to be executed (each run provides the variation of time of all sensors for an unlimited amount of time and corresponds to a single aircraft flight), divide them into two groups of the same size, and store the results for later use. These values, called $\Upsilon_{i,A}$ and $\Upsilon_{j,F}$, are, respectively, the *aircraft seeds* and the *flight seeds*, where *i* is the aircraft number representing given fixed error realizations (fixed error contributions vary from aircraft to aircraft but are constant for all flights of that aircraft), and *j* is the trajectory number representing run-to-run and in-run error realizations (run-to-run and in-run error contributions vary from one flight of a given aircraft to the next). The stored aircraft and flight seeds become the initialization seeds for each of the executions or runs, so this step does not need to be repeated.
2. Every time the simulator needs to obtain the errors generated by the different sensors (which usually correspond to a given flight), it is initialized with a given aircraft seed $\Upsilon_{i,A}$ together with a flight seed $\Upsilon_{j,F}$. As these seeds are the only inputs required for all the stochastic processes within the sensors, the results of a given run can always be repeated by employing the same two seeds.
  The selected aircraft and flight seeds are then employed to initialize two different discrete uniform distributions. One is executed five times to provide the *fixed sensor seeds* ($\upsilon_{i,A,\mathrm{ACC}}$, $\upsilon_{i,A,\mathrm{GYR}}$, $\upsilon_{i,A,\mathrm{MAG}}$, $\upsilon_{i,A,\mathrm{PLAT}}$, and $\upsilon_{i,A,\mathrm{CAM}}$), while the other is realized nine times to obtain the *run sensor seeds* ($\upsilon_{j,F,\mathrm{ACC}}$, $\upsilon_{j,F,\mathrm{GYR}}$, $\upsilon_{j,F,\mathrm{MAG}}$, $\upsilon_{j,F,\mathrm{OSP}}$, $\upsilon_{j,F,\mathrm{OAT}}$, $\upsilon_{j,F,\mathrm{TAS}}$, $\upsilon_{j,F,\mathrm{AOA}}$, $\upsilon_{j,F,\mathrm{AOS}}$, and $\upsilon_{j,F,\mathrm{GNSS}}$). These seeds hence become the initialization seeds for each of the different sensors described throughout this article.
3. Each sensor relies on either one or two standard normal distributions $N\left(0,\;1\right)$, depending on whether its error model is based exclusively on run-to-run and in-run error contributions or it also contains fixed error sources. The normal distributions of every sensor are initialized with the corresponding seeds ($\upsilon_{i,A,\mathrm{XXX}}$ and $\upsilon_{j,F,\mathrm{XXX}}$) for that sensor.
4. Upon initialization, the *fixed* normal distribution of every sensor is employed to generate all the values corresponding to scale factors, cross couplings, hard iron magnetism, and mounting errors. The *run* normal distribution in turn is employed to generate the required bias offsets.
5. Once the model has been initialized, it is able to estimate the errors generated by each sensor working at the required sensor rate. As time advances, every time a sensor is called to provide a measurement, its already initialized and used run normal distribution is called to generate the corresponding random walk increments and white noises.

### 6.2. Example: Input Parameters for a Low SWaP Fixed Wing Aircraft

This section describes the process followed by the authors to obtain the input parameters required by the various models (listed in Table 5, Table 6, Table 7 and Table 8) for the specific case of a small low SWaP fixed wing autonomous aircraft. These values constitute the default configuration of the supplied C++ implementation [6], but they can be modified by the user to reflect different hardware, mounting, or calibration procedures.

With respect to the operating frequencies, Table 13 reflects the considered values, which are all within the working range of the specific sensors described in the following paragraphs.

The gyroscope values correspond to the MEMS gyroscopes present inside the Analog Devices ADIS16488A IMU [55]. Table 14 shows its performances, which have been taken from the data sheet when possible and corrected when suspicious. A calibration process such as that described in Section 5.1 is assumed to eliminate 95% of the scale factor and cross-coupling errors.

Table 14 contains three columns of data. The left most column (“Spec”) corresponds to data taken directly from the sensor’s specifications, which become converted in the middle column (“Value”) to the parameters shown in Section 2.2,Section 2.3, Section 2.4, Section 2.5, Section 2.6, Section 2.7, Section 2.8, Section 2.9 (the conversion between bias instability and $\sigma_{u}$ uses a period of 100 s, as noted in Section 2.4). The right column (“Calibration”) contains the values after the calibration process, which reduces the scale factor and cross-coupling errors by 95%. A similar process based on the explanations of Section 2.3 and Section 2.4 should be followed by the user to obtain the parameters applicable to different gyroscopes, noting that a certain level of arbitrariness is required to account for the effects of calibration.

The accelerometer values also correspond to the MEMS accelerometers present inside the Analog Devices ADIS16488A IMU [55]. All values shown in Table 15 have been taken from the data sheet. As in the case of the gyroscopes, a calibration process as that described in Section 5.1 is assumed to eliminate 95% of the scale factor and cross-coupling errors (the conversion between bias instability and $\sigma_{u}$ uses a period of 100 s, as noted in Section 2.4). As in the previous case, a similar process should be followed by the user to obtain the parameters applicable to different accelerometers and calibration procedures.

The magnetometer values are shown in Table 16, where the white noise has been taken from [4] and the rest of the parameters correspond to the magnetometers present in the Analog Devices ADIS16488A IMU [55]. Although the value of hard and soft iron magnetism in aircraft is rather small, the authors have not been able to obtain trusted values for them. To avoid eliminating sources of error, the authors have decided to increase by 50% the values for bias offset, scale factor, and cross-coupling errors found in the literature, as shown in the compensation column (“Comp.”). As both result in a similar effect, the authors expect that the realism of the results will not be adversely affected. In the case of the bias, the authors have assigned most of the error to the fixed hard iron error $B_{\mathrm{HI},\mathrm{MAG}}$ and the remaining to the run-to-run bias offset $B_{0,\mathrm{MAG}}$.

A swinging process such as that described in Section 5.3 is assumed to eliminate 90% of the fixed error contributions, this is, the hard iron magnetism, the scale factor, and the cross-coupling error (the soft iron effect is combined with the scale factor and cross-coupling errors). The final results can be found in the rightmost column above. The user should follow a similar process to obtain the parameters applicable to each specific case. Note however that because of the influence of the hard and soft iron effects, and the intrinsic difficulty of swinging, the determination of the parameters is more arbitrary than in the case of the inertial sensors.

With respect to the GNSS receiver, the horizontal position accuracy shown in Table 17 corresponds to the U-blox NEO-M8 receiver data sheet [56], where CEP stands for circular error probability. As CEP is equivalent to 1.18 standard deviations [57], it enables the obtainment of $\sigma_{\mathrm{GNSS},\mathrm{POS},\mathrm{HOR}}$. As no vertical position accuracy is available within [56], a conservative value for $\sigma_{\mathrm{GNSS},\mathrm{POS},\mathrm{VER}}$ of twice that of $\sigma_{\mathrm{GNSS},\mathrm{POS},\mathrm{HOR}}$ has been selected. The velocity accuracy also originates at the U-blox NEO-M8 receiver data sheet [56]. Assuming that it corresponds to a per-axis error of ±0.05m/s instead of CEP, and knowing that the 50% mark of a normal distribution lies at 0.67448 standard deviations, it is possible to obtain $\sigma_{\mathrm{GNSS},\mathrm{VEL}}$.

In regards to the air data system, the $\sigma_{\mathrm{OSP}}$ value shown in Table 18 originates at the ±10 m altitude error listed in the specifications of the Aeroprobe air data system [58], which translates into ±100 Pa at a pressure altitude of 1500m. The $\sigma_{\mathrm{OAT}}$ value is taken from the Analog Devices ADT7420 temperature sensor [59]. With respect to $\sigma_{\mathrm{TAS}}$, the Aeroprobe air data system specifications [58] list a maximum airspeed error of 1m/s, which can be interpreted as 3σ, and hence results in the $\sigma_{\mathrm{TAS}}$ value included in the table. The multi-hole Pitot tube contained in the Aeroprobe air data system [58] measures both flow angles (attack and sideslip) with a maximum error of $\pm 1.0^{\circ }$. If interpreted as 3σ, this results in standard deviations $\sigma_{\mathrm{AOA}}$ and $\sigma_{\mathrm{AOS}}$ of $0.33^{\circ }$. Although never present in the data sheets, the bias offsets for all variables present in the the Section 3.3 air data system model have been set equal to the system noises to obtain further realism in the results.

Table 19 suggests appropriate values for the IMU platform and camera pose estimation errors described in Section 5.2 and Section 5.4, together with totally subjective ones for their true attitude with respect to the body frame.

## 7. Conclusions

This article presents realistic, stochastic, and customizable models for the errors generated by the sensors typically installed onboard a fixed wing aircraft. These can be used to generate pseudo-random time-stamped series of values that simulate the sensor outputs during flight as well as a series of images of the Earth’s surface that resemble what would be recorded by a camera mounted on the aircraft.

The article provides instructions and an example of how to obtain the parameters on which the models rely based exclusively on the information usually displayed in the data sheets provided by the sensors’ manufacturers, so the user can employ the values that better resemble the performances of the specific equipment being modeled. The models properly represent the stochastic nature of the different random processes involved, while ensuring that the time-stamped series of outputs generated by each sensor can be repeated if so desired. The various sensor models include the contributions of the most important sources of error, and they are intended to be used as inputs to Monte Carlo simulations that rely on the sensor outputs, such as those required to evaluate inertial, visual, and visual–inertial navigation systems.

The authors release an open-source C++ implementation of the described models.

## Figures and Tables

**Table 1:** Components of sensed trajectory.

Components	Variable	Measured by	Acronym	Rate
Specific force	${\tilde{f}}_{\mathrm{IB}}^{B}$	Accelerometers	ACC	$\Delta t_{\mathrm{SENSED}}$
Inertial angular velocity	${\tilde{\omega }}_{\mathrm{IB}}^{B}$	Gyroscopes	GYR	$\Delta t_{\mathrm{SENSED}}$
Magnetic field	${\tilde{B}}^{B}$	Magnetometers	MAG	$\Delta t_{\mathrm{SENSED}}$
Geodetic coordinates	${\tilde{x}}_{\mathrm{GDT}}$	GNSS receiver	GNSS	$\Delta t_{\mathrm{GNSS}}$
Ground velocity	${\tilde{v}}^{N}$	GNSS receiver	GNSS	$\Delta t_{\mathrm{GNSS}}$
Air pressure	$\tilde{p}$	Barometer	OSP	$\Delta t_{\mathrm{SENSED}}$
Air temperature	$\tilde{T}$	Thermometer	OAT	$\Delta t_{\mathrm{SENSED}}$
Airspeed	${\tilde{v}}_{\mathrm{TAS}}$	Pitot tube	TAS	$\Delta t_{\mathrm{SENSED}}$
Angle of attack	$\tilde{\alpha }$	Air vanes	AOA	$\Delta t_{\mathrm{SENSED}}$
Angle of sideslip	$\tilde{\beta }$	Air vanes	AOS	$\Delta t_{\mathrm{SENSED}}$
Image	I	Digital camera	CAM	$\Delta t_{\mathrm{IMG}}$

**Table 2:** Typical inertial sensor biases according to IMU grade.

IMU Grade	Accelerometer Bias [mg]	Gyroscope Bias [°/h]
Marine	0.01	0.001
Aviation	0.03–0.1	0.01
Intermediate	0.1–1	0.1
Tactical	1–10	1–100
Automotive	>10	>100

**Table 3:** Typical inertial sensor system noise according to IMU grade.

IMU Grade	Accelerometer Root PSD [m/s/h^0.5^]	Gyroscope Root PSD [°/h^0.5^]
Aviation	0.012	0.002
Tactical	0.06	0.03–0.1
Automotive	0.6	1

**Table 4:** Units for single-axis inertial sensor error sources.

Units	$B_{0}$	$\sigma_{u}$	$\sigma_{v}$	$f_{\mathrm{BW}}\left(0\right)$	$g_{\mathrm{BW}}\left(0\right)$
Accelerometer	$m/s^{2}$	$m/s^{2.5}$	$m/s^{1.5}$	m/s	m
Gyroscope	${}^{\circ }/s$	${}^{\circ }/s^{1.5}$	${}^{\circ }/s^{0.5}$	${}^{\circ }$	N/A

**Table 5:** Inertial sensor error sources.

Error		Source	Description	Seeds	
Bias Offset	$B_{0\mathrm{ACC}},\;B_{0\mathrm{GYR}}$	run-to-run	Section 2.2	$\upsilon_{j,F,\mathrm{ACC}}$, $\upsilon_{j,F,\mathrm{GYR}}$	$\Upsilon_{j,F}$
Bias Drift	$\sigma_{u\mathrm{ACC}},\;\sigma_{u\mathrm{GYR}}$	in-run	Section 2.2	$\upsilon_{j,F,\mathrm{ACC}}$, $\upsilon_{j,F,\mathrm{GYR}}$	$\Upsilon_{j,F}$
System Noise	$\sigma_{v\mathrm{ACC}},\;\sigma_{v\mathrm{GYR}}$	in-run	Section 2.2	$\upsilon_{j,F,\mathrm{ACC}}$, $\upsilon_{j,F,\mathrm{GYR}}$	$\Upsilon_{j,F}$
Scale Factor	$s_{\mathrm{ACC}},\;s_{\mathrm{GYR}}$	fixed and T	Section 2.6, Section 2.7	$\upsilon_{i,A,\mathrm{ACC}}$, $\upsilon_{i,A,\mathrm{GYR}}$	$\Upsilon_{i,A}$
Cross-Coupling	$m_{\mathrm{ACC}},\;m_{\mathrm{GYR}}$	fixed	Section 2.6, Section 2.7	$\upsilon_{i,A,\mathrm{ACC}}$, $\upsilon_{i,A,\mathrm{GYR}}$	$\Upsilon_{i,A}$
Lever Arm	$T_{\mathrm{BP}},\;\sigma_{{\hat{T}}_{\mathrm{BP}}^{B}}$	fixed	Section 2.8	$\upsilon_{i,A,\mathrm{PLAT}}$	$\Upsilon_{i,A}$
IMU Attitude	$\sigma_{\psi^{P}},\;\sigma_{\theta^{P}},\;\sigma_{\xi^{P}},\;\sigma_{{\hat{\phi }}^{\mathrm{BP}}}$	fixed	Section 2.8	$\upsilon_{i,A,\mathrm{PLAT}}$	$\Upsilon_{i,A}$

**Table 6:** Magnetometer error sources.

Error		Source	Seeds	
Hard Iron	$B_{\mathrm{HI},\mathrm{MAG}}$	fixed	$\upsilon_{i,A,\mathrm{MAG}}$	$\Upsilon_{i,A}$
Bias Offset	$B_{0,\mathrm{MAG}}$	run-to-run	$\upsilon_{j,F,\mathrm{MAG}}$	$\Upsilon_{j,F}$
System Noise	$\sigma_{v,\mathrm{MAG}}$	in-run	$\upsilon_{j,F,\mathrm{MAG}}$	$\Upsilon_{j,F}$
Scale Factor	$s_{\mathrm{MAG}}$	fixed	$\upsilon_{i,A,\mathrm{MAG}}$	$\Upsilon_{i,A}$
Cross Coupling	$m_{\mathrm{MAG}}$	fixed	$\upsilon_{i,A,\mathrm{MAG}}$	$\Upsilon_{i,A}$

**Table 7:** GNSS receiver error sources.

Error		Source	Seeds	
Bias Offset	$B_{0,\mathrm{GNSS},\mathrm{ION}}$	run-to-run	$\upsilon_{j,F,\mathrm{GNSS}}$	$\Upsilon_{j,F}$
System Noise	$\sigma_{\mathrm{GNSS},\mathrm{POS}},\;\sigma_{\mathrm{GNSS},\mathrm{VEL}},\;\sigma_{\mathrm{GNSS},\mathrm{ION}}$	in-run	$\upsilon_{j,F,\mathrm{GNSS}}$	$\Upsilon_{j,F}$

**Table 8:** Air data sensor error sources.

Error		Source	Seeds	
Bias Offset	$B_{0\mathrm{OSP}},B_{0\mathrm{OAT}},B_{0\mathrm{TAS}},B_{0\mathrm{AOA}},B_{0\mathrm{AOS}}$	run-to-run	$\upsilon_{j,F,\mathrm{OSP}},\upsilon_{j,F,\mathrm{OAT}}$	$\Upsilon_{j,F}$
System Noise	$\sigma_{\mathrm{OSP}},\sigma_{\mathrm{OAT}},\sigma_{\mathrm{TAS}},\sigma_{\mathrm{AOA}},\sigma_{\mathrm{AOS}}$	in-run	$\upsilon_{j,F,\mathrm{TAS}},\upsilon_{j,F,\mathrm{AOA}},\upsilon_{j,F,\mathrm{AOS}}$	

**Table 9:** Camera parameters.

Parameter	Symbol	Unit
Focal length	f	mm
Image width	$S_{H}$	px
Image height	$S_{V}$	px
Pixel size	$s_{\mathrm{PX}}$	mm/px
Principal point horizontal location	$c_{1}^{\mathrm{IMG}}$	px
Principal point vertical location	$c_{2}^{\mathrm{IMG}}$	px
Horizontal field of view	$\Theta_{H}$	${}^{\circ }$
Vertical field of view	$\Theta_{V}$	${}^{\circ }$

**Table 10:** Results of calibration process.

Estimation	#	Coefficients
${\hat{M}}_{\mathrm{ACC}}$	6	$\left\{{\hat{s}}_{\mathrm{ACC},i},\;{\hat{m}}_{\mathrm{ACC},\mathrm{ij}}\right\}$
${\hat{M}}_{\mathrm{GYR}}$	9	$\left\{{\hat{s}}_{\mathrm{GYR},i},\;{\hat{m}}_{\mathrm{GYR},\mathrm{ij}}\right\}$
${\hat{B}}_{0\mathrm{ACC}}$	3	$B_{0\mathrm{ACC}}\;{\hat{N}}_{u0,\mathrm{ACC},i}$
${\hat{B}}_{0\mathrm{GYR}}$	3	$B_{0\mathrm{GYR}}\;{\hat{N}}_{u0,\mathrm{GYR},i}$

**Table 11:** Results of swinging process.

Estimation	#	Coefficients
${\hat{M}}_{\mathrm{MAG}}$	9	$\left\{{\hat{s}}_{\mathrm{MAG},i},\;{\hat{m}}_{\mathrm{MAG},\mathrm{ij}}\right\}$
${\hat{B}}_{\mathrm{HI},\mathrm{MAG}}$	3	${\hat{B}}_{\mathrm{HI},\mathrm{MAG},i}$
${\hat{B}}_{0,\mathrm{MAG}}$	3	$B_{0,\mathrm{MAG}}\;{\hat{N}}_{u0,\mathrm{MAG},i}$

**Table 12:** Sensor seeds.

Type		Error Sources	Seeds
Aircraft	i	fixed	$\upsilon_{i,A,\mathrm{ACC}},\;\upsilon_{i,A,\mathrm{GYR}},\;\upsilon_{i,A,\mathrm{MAG}},\;\upsilon_{i,A,\mathrm{PLAT}},\;\upsilon_{i,A,\mathrm{CAM}}$
Flight	j	run-to-run and in-run	$\upsilon_{j,F,\mathrm{ACC}},\;\upsilon_{j,F,\mathrm{GYR}},\;\upsilon_{j,F,\mathrm{MAG}},\;\upsilon_{j,F,\mathrm{OSP}},\;\upsilon_{j,F,\mathrm{OAT}}$
			$\upsilon_{j,F,\mathrm{TAS}},\;\upsilon_{j,F,\mathrm{AOA}},\;\upsilon_{j,F,\mathrm{AOS}},\;\upsilon_{j,F,\mathrm{GNSS}}$

**Table 13:** Example of frequencies of the different sensors.

Discrete Time	Frequency	Rate
$t_{t}=t\cdot \Delta t_{\mathrm{TRUTH}}$	500Hz	0.002s
$t_{s}=s\cdot \Delta t_{\mathrm{SENSED}}$	100Hz	0.01s
$t_{i}=i\cdot \Delta t_{\mathrm{IMG}}$	10Hz	0.1s
$t_{g}=g\cdot \Delta t_{\mathrm{GNSS}}$	1Hz	1s

**Table 14:** Example for gyroscopes performance values.

GYR	Spec	Unit	Variable	Value	Calibration	Unit
In-Run Bias Stability (1 σ)	5.10	${}^{\circ }/h$	$\sigma_{u\mathrm{GYR}}$	$1.42\times 10^{-4}$	$1.42\times 10^{-4}$	${}^{\circ }/s^{1.5}$
Angle Random Walk (1 σ)	0.26	${}^{\circ }/h^{0.5}$	$\sigma_{v\mathrm{GYR}}$	$4.30\times 10^{-3}$	$4.30\times 10^{-3}$	${}^{\circ }/s^{0.5}$
Nonlinearity ^1^	0.01	%	$s_{\mathrm{GYR}}$	$3.00\times 10^{-4}$	$1.50\times 10^{-5}$	-
Misalignment	±0.05	${}^{\circ }$	$m_{\mathrm{GYR}}$	$8.70\times 10^{-4}$	$4.35\times 10^{-5}$	-
Bias Repeatability (1 σ)	±0.2	${}^{\circ }/s$	$B_{0\mathrm{GYR}}$	$2.00\times 10^{-1}$	$2.00\times 10^{-1}$	${}^{\circ }/s$

^1^ The 0.01% scale factor error obtined in [55] is considered too optimistic and hence modified to 0.03% = 3.00 × 10^−4^.

**Table 15:** Example for accelerometers’ performance values.

ACC	Spec	Unit	Variable	Value	Calibration	Unit
In-Run Bias Stability (1 σ)	0.07	mg	$\sigma_{u\mathrm{ACC}}$	$6.86\times 10^{-5}$	$6.86\times 10^{-5}$	$m/s^{2.5}$
Velocity Random Walk (1 σ)	0.029	$m/s/h^{0.5}$	$\sigma_{v\mathrm{ACC}}$	$4.83\times 10^{-4}$	$4.83\times 10^{-4}$	$m/s^{1.5}$
Nonlinearity	0.1	%	$s_{\mathrm{ACC}}$	$1.00\times 10^{-3}$	$5.00\times 10^{-5}$	-
Misalignment	±0.035	${}^{\circ }$	$m_{\mathrm{ACC}}$	$6.11\times 10^{-4}$	$3.05\times 10^{-5}$	-
Bias Repeatability (1 σ)	±16	mg	$B_{0\mathrm{ACC}}$	$1.57\times 10^{-1}$	$1.57\times 10^{-1}$	$m/s^{2}$

**Table 16:** Example for magnetometers’ performance values.

MAG	Spec	Unit	Variable	Value	Comp.	Swinging	Unit
Output Noise	5	$\mathrm{nT}\cdot s^{0.5}$	$\sigma_{v,\mathrm{MAG}}$	$5.00\times 10^{0}$	$5.00\times 10^{0}$	$5.00\times 10^{0}$	$\mathrm{nT}\cdot s^{0.5}$
Nonlinearity	0.5	%	$s_{\mathrm{MAG}}$	$5.00\times 10^{-3}$	$7.50\times 10^{-3}$	$7.50\times 10^{-4}$	-
Misalignment	±0.35	${}^{\circ }$	$m_{\mathrm{MAG}}$	$6.11\times 10^{-3}$	$9.16\times 10^{-3}$	$9.16\times 10^{-4}$	-
Bias (1 σ)	±1500	nT	$B_{\mathrm{HI},\mathrm{MAG}}$	$1.50\times 10^{3}$	$1.75\times 10^{3}$	$1.75\times 10^{2}$	nT
Repeatability			$B_{0,\mathrm{MAG}}$		$5.00\times 10^{2}$	$5.00\times 10^{2}$	nT

**Table 17:** Example of GNSS receiver performance values.

GNSS	Spec	Unit	Variable	Value	Unit
Horizontal position accuracy (CEP 50%)	2.50	m	$\sigma_{\mathrm{GNSS},\mathrm{POS},\mathrm{HOR}}$	$2.12\times 10^{0}$	m
Vertical position accuracy (CEP 50%)	N/A		$\sigma_{\mathrm{GNSS},\mathrm{POS},\mathrm{VER}}$	$4.25\times 10^{0}$	m
Ionospheric random walk 1/60Hz	N/A		$\sigma_{\mathrm{GNSS},\mathrm{ION}}$	$1.60\times 10^{-1}$	m
Ionospheric bias offset	N/A		$B_{0,\mathrm{GNSS},\mathrm{ION}}$	$8.00\times 10^{0}$	m
Velocity accuracy (50%)	0.05	m/s	$\sigma_{\mathrm{GNSS},\mathrm{VEL}}$	$7.41\times 10^{-2}$	m/s

**Table 18:** Example of air data system performance values.

Air Data System	Spec	Unit	Variable	Value	Unit
Altitude Error	±10	m	$\sigma_{\mathrm{OSP}}$	$1.00\times 10^{2}$	Pa
			$B_{0\mathrm{OSP}}$	$1.00\times 10^{2}$	Pa
Temperature Error (3σ)	±0.15	K	$\sigma_{\mathrm{OAT}}$	$5.00\times 10^{-2}$	K
			$B_{0\mathrm{OAT}}$	$5.00\times 10^{-2}$	K
Airspeed Error (max)	1	m/s	$\sigma_{\mathrm{TAS}}$	$3.33\times 10^{-1}$	m/s
			$B_{0\mathrm{TAS}}$	$3.33\times 10^{-1}$	m/s
Flow Angle Error (max)	±1.0	${}^{\circ }$	$\sigma_{\mathrm{AOA}}$	$3.33\times 10^{-1}$	${}^{\circ }$
			$B_{0\mathrm{AOA}}$	$3.33\times 10^{-1}$	${}^{\circ }$
Flow Angle Error (max)	±1.0	${}^{\circ }$	$\sigma_{\mathrm{AOS}}$	$3.33\times 10^{-1}$	${}^{\circ }$
			$B_{0\mathrm{AOS}}$	$3.33\times 10^{-1}$	${}^{\circ }$

**Table 19:** Example for IMU and camera mounting accuracy values.

Concept	Variable	Value	Unit	Variable	Value	Unit
True Yaw Error	$\sigma_{\psi^{P}}$	0.5	${}^{\circ }$	$\sigma_{\psi^{C}}$	0.1	${}^{\circ }$
True Pitch Error	$\sigma_{\theta^{P}}$	2.0	${}^{\circ }$	$\sigma_{\theta^{C}}$	0.1	${}^{\circ }$
True Bank Error	$\sigma_{\xi^{P}}$	0.1	${}^{\circ }$	$\sigma_{\xi^{C}}$	0.1	${}^{\circ }$
Position Estimation Error	$\sigma_{{\hat{T}}_{\mathrm{BP}}^{B}}$	0.01	m	$\sigma_{{\hat{T}}_{\mathrm{BC}}^{B}}$	0.002	m
Attitude Estimation Error	$\sigma_{{\hat{\phi }}^{\mathrm{BP}}}$	0.03	${}^{\circ }$	$\sigma_{{\hat{\phi }}^{\mathrm{BC}}}$	0.01	${}^{\circ }$

**Figure A1:**
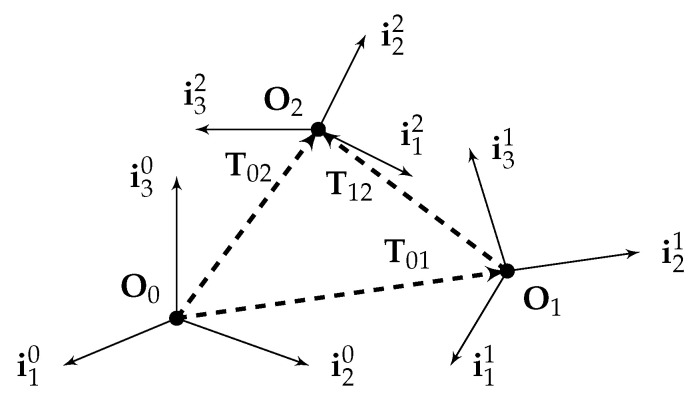
Reference system for combination of movements.

## Data Availability

An open source C++ implementation of the described models can be found at [6]. Its execution generates pseudo-random time-stamped series of the errors introduced by the different sensors.

## Abbreviations

The following abbreviations are used in this manuscript:

ACC	ACCelerometer
AOA	Angle of Attack
AOS	Angle of Sideslip
CAM	CAMera
CEP	Circular Error Probability
GLONASS	GLObal NAvigation Satellite System
GNC	Guidance, Navigation, and Control
GNSS	Global Navigation Satellite System
GPS	Global Positioning System
GYR	GYRoscope
IMU	Inertial Measurement Unit
ISO	International Organization for Standardization
MAG	MAGnetometer
MEMS	Micromachined ElectroMechanical System
NED	North–East–Down
OAT	Outside Air Temperature
OSP	Outside Static Pressure
PSD	Power Spectral Density
SWaP	Size, Weight, and Power
TAS	True Air Speed

## Appendix A. Motion of Multiple Rigid Bodies

The equations employed in this article make use of positions, velocities, and accelerations (both linear and angular) that refer to different reference systems or rigid bodies, which are in continuous motion (translation and rotation) among themselves. Their relationships are obtained in this appendix based on the three reference systems shown in Figure A1: an inertial reference system $F_{0}\left\{0_{0},\;i_{1}^{0},\;i_{2}^{0},\;i_{3}^{0}\right\}$ and two non-inertial reference systems $F_{1}\left\{0_{1},\;i_{1}^{1},\;i_{2}^{1},\;i_{3}^{1}\right\}$ and $F_{2}\left\{0_{2},\;i_{1}^{2},\;i_{2}^{2},\;i_{3}^{2}\right\}$, where $\left(T_{01},\;v_{01},\;a_{01}\right)$ are the position, linear velocity, and linear acceleration of the origin of $F_{1}$ with respect to $F_{0}$, $\left(T_{02},\;v_{02},\;a_{02}\right)$ are those of the origin of $F_{2}$ with respect to $F_{0}$, and $\left(T_{12},\;v_{12},\;a_{12}\right)$ are those of the origin of $F_{2}$ with respect to $F_{1}$. Similarly, $\left(\omega_{01},\;\alpha_{01}\right)$ are the angular velocity and angular acceleration of $F_{1}$ with respect to $F_{0}$, $\left(\omega_{02},\;\alpha_{02}\right)$ are those of $F_{2}$ with respect to $F_{0}$, and $\left(\omega_{12},\;\alpha_{12}\right)$ are those of $F_{2}$ with respect to $F_{1}$. Let us also consider that $R_{01}$, $R_{02}$, and $R_{12}$ are the rotation matrices among the three different rigid bodies.

### Appendix A.1. Composition of Position

The relationship between the linear position vectors $T_{02}$, $T_{12}$, and $T_{01}$ can be established by vectorial arithmetics when expressed in the same reference frame or by coordinate transformation [45] when not so:

(A1) $$T_{02}^{0}=T_{12}^{0}+T_{01}^{0}=R_{01}\;T_{12}^{1}+T_{01}^{0}$$

### Appendix A.2. Composition of Linear Velocity

The derivation with time of (A1) results in:

(A2) $${\dot{T}}_{02}^{0}={\dot{R}}_{01}\;T_{12}^{1}+R_{01}\;{\dot{T}}_{12}^{1}+{\dot{T}}_{01}^{0}$$

The use of the relationship between the rotation matrix and its time derivative [45] results in (note that the wide hat ˂$\hat{\cdot }$˃ refers to the skew-symmetric form of a vector):

(A3) $${\dot{T}}_{02}^{0}=R_{01}\;{\hat{\omega }}_{01}^{1}\;T_{12}^{1}+R_{01}\;{\dot{T}}_{12}^{1}+{\dot{T}}_{01}^{0}$$

Reordering and replacing the position time derivatives with their respective velocities results in the relationship between the linear velocity vectors $v_{02}$, $v_{12}$, and $v_{01}$ expressed in the inertial frame $F_{0}$:

(A4) $$\begin{array}{ccc} v_{02}^{0} & = & R_{01}\;v_{12}^{1}+v_{01}^{0}+R_{01}\;{\hat{\omega }}_{01}^{1}\;T_{12}^{1} \end{array}$$

(A5) $$\begin{array}{ccc} v_{02}^{0} & = & v_{12}^{0}+v_{01}^{0}+{\hat{\omega }}_{01}^{0}\;T_{12}^{0} \end{array}$$

### Appendix A.3. Composition of Linear Acceleration

The derivation with time of (A4) results in:

(A6) $${\dot{v}}_{02}^{0}={\dot{R}}_{01}\;v_{12}^{1}+R_{01}\;{\dot{v}}_{12}^{1}+{\dot{v}}_{01}^{0}+{\dot{R}}_{01}\;{\hat{\omega }}_{01}^{1}\;T_{12}^{1}+R_{01}\;{\dot{\hat{\omega }}}_{01}^{1}\;T_{12}^{1}+R_{01}\;{\hat{\omega }}_{01}^{1}\;{\dot{T}}_{12}^{1}$$

Replacing the rotation matrix time derivative results in:

(A7) $${\dot{v}}_{02}^{0}=R_{01}\;{\hat{\omega }}_{01}^{1}\;v_{12}^{1}+R_{01}\;{\dot{v}}_{12}^{1}+{\dot{v}}_{01}^{0}+R_{01}\;{\hat{\omega }}_{01}^{1}\;{\hat{\omega }}_{01}^{1}\;T_{12}^{1}+R_{01}\;{\dot{\hat{\omega }}}_{01}^{1}\;T_{12}^{1}+R_{01}\;{\hat{\omega }}_{01}^{1}\;{\dot{T}}_{12}^{1}$$

Reordering, replacing the position, linear velocity, and angular velocity time derivatives with their respective velocities, linear accelerations and angular accelerations, results in the relationship between the linear acceleration vectors $a_{02}$, $a_{12}$, and $a_{01}$ expressed in the inertial frame $F_{0}$:

(A8) $$\begin{array}{ccc} a_{02}^{0} & = & R_{01}\;a_{12}^{1}+\left(a_{01}^{0}+R_{01}\;{\hat{\alpha }}_{01}^{1}\;T_{12}^{1}+R_{01}\;{\hat{\omega }}_{01}^{1}\;{\hat{\omega }}_{01}^{1}\;T_{12}^{1}\right)+2\;R_{01}\;{\hat{\omega }}_{01}^{1}\;v_{12}^{1} \end{array}$$

(A9) $$\begin{array}{ccc} a_{02}^{0} & = & a_{12}^{0}+\left(a_{01}^{0}+{\hat{\alpha }}_{01}^{0}\;T_{12}^{0}+{\hat{\omega }}_{01}^{1}\;{\hat{\omega }}_{01}^{1}\;T_{12}^{0}\right)+2\;{\hat{\omega }}_{01}^{1}\;v_{12}^{0} \end{array}$$

The term on the left-hand side is called absolute acceleration, while the three right-hand side terms are usually named relative, transport, and Coriolis accelerations, respectively.

### Appendix A.4. Composition of Angular Velocity

The relationship among the different frames angular velocities is given by the rotation matrix composition rule [45], which can be derivated with respect to time:

(A10) $$R_{02}=R_{01}\;R_{12}\;\to \;{\dot{R}}_{02}={\dot{R}}_{01}\;R_{12}+R_{01}\;{\dot{R}}_{12}$$

Replacing the rotation matrix time derivatives results in:

(A11) $$R_{02}\;{\hat{\omega }}_{02}^{2}={\hat{\omega }}_{01}^{0}\;R_{01}\;R_{12}+R_{01}\;R_{12}\;{\hat{\omega }}_{12}^{2}={\hat{\omega }}_{01}^{0}\;R_{02}+R_{02}\;{\hat{\omega }}_{12}^{2}=R_{02}\;{\hat{\omega }}_{01}^{2}+R_{02}\;{\hat{\omega }}_{12}^{2}$$

The relationship among the angular velocity vectors $\omega_{02}$, $\omega_{12}$, and $\omega_{01}$ is hence the following:

(A12) $$\omega_{02}^{0}=\omega_{12}^{0}+\omega_{01}^{0}=R_{01}\;\omega_{12}^{1}+\omega_{01}^{0}$$

### Appendix A.5. Composition of Angular Acceleration

The derivation with time of (A12) results in:

(A13) $${\dot{\omega }}_{02}^{0}={\dot{R}}_{01}\;\omega_{12}^{1}+R_{01}\;{\dot{\omega }}_{12}^{1}+{\dot{\omega }}_{01}^{0}$$

Replacing the rotation matrix time derivatives results in:

(A14) $${\dot{\omega }}_{02}^{0}=R_{01}\;{\hat{\omega }}_{01}^{1}\;{\hat{\omega }}_{12}^{1}+R_{01}\;{\dot{\omega }}_{12}^{1}+{\dot{\omega }}_{01}^{0}$$

Reordering, replacing the angular velocity time derivatives with their respective angular accelerations, results in the relationship between the angular acceleration vectors $\alpha_{02}$, $\alpha_{12}$, and $\alpha_{01}$ expressed in the inertial frame $F_{0}$:

(A15) $$\begin{array}{ccc} \alpha_{02}^{0} & = & R_{01}\;\alpha_{12}^{1}+\alpha_{01}^{0}+R_{01}\;{\hat{\omega }}_{01}^{1}\;{\hat{\omega }}_{12}^{1} \end{array}$$

(A16) $$\begin{array}{ccc} \alpha_{02}^{0} & = & \alpha_{12}^{0}+\alpha_{01}^{0}+{\hat{\omega }}_{01}^{0}\;{\hat{\omega }}_{12}^{0} \end{array}$$

### Appendix A.6. Summary of Compositions

The final expressions of the compositions above (A1), (A5), (A9), (A12), and (A16) are all expressed in the inertial frame $F_{0}$, but they are also valid in any other frame as long as all its components are converted into that frame (note that it is not the same to compute a time derivative (velocity, acceleration, or angular acceleration) in the inertial frame and then convert it into a different frame than to directly compute the derivative in a non-inertial frame):

(A17) $$\begin{array}{ccc} T_{02} & = & T_{12}+T_{01} \end{array}$$

(A18) $$\begin{array}{ccc} v_{02} & = & v_{12}+v_{01}+{\hat{\omega }}_{01}\;T_{12} \end{array}$$

(A19) $$\begin{array}{ccc} a_{02} & = & a_{12}+\left(a_{01}+{\hat{\alpha }}_{01}\;T_{12}+{\hat{\omega }}_{01}\;{\hat{\omega }}_{01}\;T_{12}\right)+2\;{\hat{\omega }}_{01}\;v_{12} \end{array}$$

(A20) $$\begin{array}{ccc} \omega_{02} & = & \omega_{12}+\omega_{01} \end{array}$$

(A21) $$\begin{array}{ccc} \alpha_{02} & = & \alpha_{12}+\alpha_{01}+{\hat{\omega }}_{01}\;\omega_{12} \end{array}$$
